# Choline pelargonate treatments decreased downy mildew and powdery mildew symptoms with negligible effects on grapevine phyllosphere micro-organisms

**DOI:** 10.1093/femsec/fiag082

**Published:** 2026-07-24

**Authors:** Sofia Montanari, Martin Hartmann, Andrea Nesler, Oscar Giovannini, Claudia M O Longa, Michele Perazzolli

**Affiliations:** Center Agriculture Food Environment (C3A), University of Trento, Via E. Mach 1, 38098 San Michele all’Adige, Trento, Italy; Research and Innovation Centre, Fondazione Edmund Mach, Via E. Mach 1, 38098 San Michele all’Adige, Trento, Italy; Institute of Agricultural Sciences, Department of Environmental Systems Science, ETH Zurich, Universitätsstrasse 2, 8092 Zürich, Switzerland; Bi-PA nv (Biological Products for Agriculture), Technologielaan 7, 1840 Londerzeel, Belgium; Research and Innovation Centre, Fondazione Edmund Mach, Via E. Mach 1, 38098 San Michele all’Adige, Trento, Italy; Research and Innovation Centre, Fondazione Edmund Mach, Via E. Mach 1, 38098 San Michele all’Adige, Trento, Italy; Center Agriculture Food Environment (C3A), University of Trento, Via E. Mach 1, 38098 San Michele all’Adige, Trento, Italy; Research and Innovation Centre, Fondazione Edmund Mach, Via E. Mach 1, 38098 San Michele all’Adige, Trento, Italy

**Keywords:** amplicon sequencing, grapevine microbiome, biofungicide, gene expression, antifungal activity, choline carboxylates, trimethyl-ethanolamine nonanoate, induced resistance

## Abstract

Grapevine is an important crop worldwide, but most cultivars are susceptible to downy mildew and powdery mildew. This study aimed to evaluate the efficacy of choline pelargonate (CP) against grapevine downy mildew and powdery mildew under controlled and field conditions, and to assess its effects on phyllosphere micro-organisms. CP decreased the severity of both pathogens under greenhouse and field conditions. In greenhouse trials, 16 mM CP showed efficacy comparable to copper and sulfur applied at label-recommended concentrations as reference fungicides in organic viticulture. CP showed direct inhibitory activity against both pathogens, and slightly induced defense-related grapevine genes (*CHIT-3, OSM-2*, and *PR-1*). Field experiments corroborated the efficacy of CP against powdery mildew and downy mildew severity on leaves and bunches. Microbial community analysis revealed that plant compartment, vineyard location, and sampling time were key drivers of microbial community structure, while treatments showed negligible effects on microbial alpha-diversity and beta-diversity of grapevine leaves and bunches. CP treatment partially modified the relative abundances of some bacterial and fungal taxa, while reference fungicides caused broad changes across multiple genera. CP showed promising antifungal activity with minimal effects on phyllosphere micro-organisms, supporting its potential as a sustainable disease management approach that preserves indigenous microbial communities.

## Teaser

Choline pelargonate reduced downy mildew and powdery mildew severity with negligible effects on grapevine phyllosphere micro-organisms.

## Introduction

Grapevine (*Vitis vinifera*) is one of the most economically important perennial crops worldwide, but most grapevine cultivars are susceptible to a wide range of pathogens that can negatively impact plant growth, fruit production, and fruit quality (Armijo et al. 2016). In particular, downy mildew and powdery mildew are two of the most devastating diseases that affect grapevines (Pirrello et al. 2019, Koledenkova et al. 2022). Grapevine downy mildew is caused by the biotrophic oomycete *Plasmopara viticola* that can infect all green tissues (e.g. leaves, bunches, and shoots) in the presence of surface wetness and warm temperatures, causing yellow-green lesions on leaves (called oil spots) and brown symptoms on bunches (Koledenkova et al. 2022). Grapevine powdery mildew is caused by the biotrophic ascomycete *Erysiphe necator* that can colonize the epidermal layer of green tissues under warm and humid conditions, forming infected areas covered with a whitish-gray dusty layer of conidia (Pirrello et al. 2019).

Frequent fungicide applications (e.g. every 7–10 days, depending on phenology and weather conditions in seasons and regions with high disease pressure) are necessary to prevent losses caused by downy mildew and powdery mildew infections (Gadoury et al. 2012, Giovannini et al. 2022). For example, traditional products for downy mildew control are copper-based products, dithiocarbamates, and phthalimides, (Gessler et al. 2011, Peng et al. 2024). Additional active ingredients used against grapevine downy mildew are famoxadone, fluopicolide, mandipropamid (Peng et al. 2024), and systemic fungicides (e.g. azoxystrobin, cymoxanil, cyazofamid, dimethomorph, metalaxyl, famoxadone, fluopicolide, and fosetyl-aluminium) (Koledenkova et al. 2022, Clippinger et al. 2024, Peng et al. 2024). Moreover, powdery mildew control is commonly based on the application of sterol biosynthesis inhibitors (e.g. difenoconazole, fenbuconazole, penconazole), quinone inhibitors (e.g. azoxystrobin and kresoxim-methyl), cyflufenamid, metrafenone, spiroxamine, and sulfur (Kunova et al. 2021). The overuse of pesticides raises concerns about potential negative effects on human health and the environment (Fantke et al. 2012), leading several countries to develop regulations aimed at reducing fungicide applications (Hillocks 2012, Skevas et al. 2013). For example, copper use is limited in the European Union (EU) up to a maximum of 28 kg/ha over seven year period (Commission 2018), and further restrictions are expected in the future at the national level and by farmer associations (Cabús et al. 2017, Tamm et al. 2022). Moreover, several fungicides are under scrutiny for substitution in the EU, including active substances that are currently used against downy mildew and powdery mildew (e.g. cymoxanil, metalaxyl, fluopicolide and triazoles) (Commission 2015). Thus, there is a growing interest in developing sustainable alternatives for downy mildew and powdery mildew control in viticulture.

Innovative approaches under development for downy mildew control involve the use of biocontrol agents (e.g. *Trichoderma harzianum, Bacillus* spp., *Lysobacter capsici, Saccharomyces cerevisiae*, and *Streptomyces* spp.), plant extracts (e.g. *Artemisia* spp., *Larix* spp., *Origanum vulgare*, and *Salvia officinalis*), inorganic compounds (e.g. potassium bicarbonate, silicon nanoparticles, and 3-aminobutyric acid), and organic compounds (e.g. chitosan and laminarin) (Clippinger et al. 2024, Nadalini and Puopolo 2024, Peng et al. 2024). Likewise, emerging strategies for powdery mildew control include the use of potassium bicarbonate, biocontrol agents (e.g. *Ampelomyces quisqualis*), and organic compounds (e.g. laminarin, chitin, chitosan, and COS-OGA) (Pertot et al. 2017). However, care should be taken to verify if emerging alternatives could pose harmful effects on non-target micro-organisms and microbial ecosystems (Clippinger et al. 2024). Amplicon sequencing analyses of the grapevine phyllosphere (leaves, flowers, and grape berry surface) demonstrated that the relative abundances of bacterial and fungal communities are affected by agronomic practices, plant compartment, grape cultivar, time point of sampling, and vineyard location (Morgan et al. 2017). In particular, fungicide treatments can slightly influence the structure of bacterial and fungal communities of the grapevine phyllosphere (Perazzolli et al. 2014, Escribano-Viana et al. 2018, Gobbi et al. 2020, Perazzolli et al. 2020, Mondello et al. 2022, He et al. 2024, Yang et al. 2024). For example, grapevine treatments with a biocontrol agent (*Lysobacter capsici* AZ78) or a chemical fungicide (penconazole) can affect the relative abundance of some bacterial taxa (e.g. *Alcaligenaceae, Bacillaceae, Enterobacteriaceae, Methylobacteriaceae, Microbacteriaceae*, and *Moraxellaceae*) and fungal taxa (e.g. *Microstromataceae* and *Tremellaceae*), although the vineyard location strongly affects richness and diversity of leaf-associated microbial communities (Perazzolli et al. 2014). Likewise, copper and *Lactobacillus plantarum* MW-1 treatments are known to alter the relative abundance of a few bacterial taxa (e.g. *Lactobacillaceae*) and fungal taxa (e.g. *Botryosphaeriaceae, Filobasidiales, Leptosphaeriaceae*) on grapevine leaves, whereas the time point of sampling acts as the major driver of changes in the microbial community structure (Gobbi et al. 2020). In other studies, bacterial and fungal populations were not affected by conventional fungicides applied against powdery mildew on grapevine leaves (Yang et al. 2024) and a copper-based fungicide against esca disease on wood (Mondello et al. 2022), corroborating the negligible effects of fungicide treatments on grapevine-associated microbial communities. However, other fungicides (e.g. pyraclostrobin and myclobutanil) can partially affect the diversity and composition of bacterial communities of grapevine leaves (He et al. 2024), while a biocontrol agent (e.g. *Bacillus subtilis* OCASB12) (He et al. 2024) and a sugar-based fungicide (tagatose) (Perazzolli et al. 2020) have minimal effects on some phytopathogens (e.g. *Erysiphe* spp. and *Alternaria* spp.) and potential beneficial micro-organisms (e.g. *Aureobasidium* spp., *Chaetomium* spp., *Issatchenkia* spp., *Hygrophorus* spp., and *Pseudomonas* spp.). Likewise, treatments with a biocontrol agent (*Bacillus subtilis* QST713) showed negligible effects on the wine microbiota, whereas the chemical fungicide (fenhexamid) caused a reduction of bacterial richness and diversity (Escribano-Viana et al. 2018), suggesting differential effects of chemical and biological fungicides on non-target micro-organisms. Thus, the understanding of the possible effects of new fungicides on phyllosphere micro-organisms is essential for the further development of sustainable plant protection products.

Choline pelargonate (CP; also known as trimethyl-ethanolamine nonanoate or choline nonanoate) is a novel formulation of pelargonic acid in the form of a choline salt, proposed as a sustainable fungicide (De Saegher et al. 2019). Pelargonic acid (also known as nonanoic acid) is a free fatty acid, naturally present at low concentrations in foods (e.g. fruits, vegetables, milk, and beef), and it is used as an herbicide in concentrated formulations (Ciriminna et al. 2019). Moreover, choline is considered safe by the Food and Drug Administration (U.S.A.) and the European Food Safety Agency (Li et al. 2022), and is a micronutrient of human cells (Penry and Manore 2008). CP belongs to the subclass of ionic liquids known as choline carboxylates, a group of biocompatible substances that have been studied as solvents in the cosmetic, food, and pharmaceutical industries (Rengstl et al. 2014, Elhi et al. 2020). CP can inhibit mycelial growth and spore germination of *Botrytis cinerea* and *Phytophthora infestans*, causing the leakage of electrolytes and nucleic acids with negative impacts on membrane functionality and lipid metabolism *in vitro*, suggesting promising inhibitory activity against oomycete and ascomycete phytopathogens (Montanari et al. 2025). However, the efficacy of CP against plant pathogens has not yet been investigated under field conditions, and its possible impacts on plant-associated microbial communities have not yet been evaluated. This study aimed to assess the efficacy of CP against grapevine downy mildew and powdery mildew under controlled and field conditions, and to evaluate its potential side effects on the grapevine phyllosphere microbiota.

## Materials and methods

### Biological material and artificial inoculations under greenhouse conditions

Two-year-old plants (*V. vinifera* cultivar Pinot Noir) were grown in 2.5 l pots containing a mixture of peat and pumice (3 : 1; GR Intensivo, Tercomposti, Calvisano, Italy) under greenhouse conditions with a 16 h light / 8 h dark photoperiod at 25 ± 1°C and 70 ± 10% relative humidity (RH) as previously described (Perazzolli et al. 2012).

A *P. viticola* population was collected from an untreated vineyard in the Trentino region (northern Italy) and maintained on *V. vinifera* plants (cultivar Pinot Noir) through subsequent inoculations under greenhouse conditions (Perazzolli et al. 2012). To obtain *P. viticola* inoculum, plants exhibiting disease symptoms were incubated overnight in the dark at 97 ± 3% relative humidity (RH) to promote pathogen sporulation, and sporangia were collected by washing the abaxial leaf surfaces bearing freshly sporulating lesions with cold (4°C) distilled water. The inoculum concentration was assessed with a hemacytometer under a light microscope (Eclipse 80i, Nikon, Tokyo, Japan) and adjusted to 2.5 × 10^5^ sporangia/ml (plant inoculation in efficacy and translaminar trials) or 1 × 10^6^ sporangia/ml (viability tests of sporangia and zoospores).

The inoculum of *E. necator* was obtained from infected leaves of an untreated vineyard (Trentino region, northern Italy) and maintained on *V. vinifera* plants (cultivar Pinot Noir) through subsequent inoculations under greenhouse conditions (25 ± 1°C and 70 ± 10% RH) as described by Nesler et al. (2015). To obtain *E. necator* inoculum for efficacy trials, conidia were collected from freshly sporulating leaves by washing the adaxial leaf surfaces with distilled water. The inoculum concentration was assessed with a hemacytometer under a light microscope (Eclipse 80i, Nikon), adjusted to 2 × 10^4^ conidia/ml, and immediately used for spray inoculation as described for zucchini powdery mildew (Cappelletti et al. 2017). To prepare *E. necator* inoculum for conidial germination trials, conidia were brushed gently from infected young leaves carrying fresh sporulation of powdery mildew using a paintbrush directly on leaf disks (Nesler et al. 2015).

The stock solution of CP [41% (w/v); CAS number 10246–69–2] was provided by Bi-PA nv. (Londerzeel, Belgium) and diluted in water for greenhouse and field trials.

### Assessment of choline pelargonate efficacy against downy mildew on grapevine leaf discs

The efficacy against downy mildew on grapevine leaf disks was assessed as described by Smaldone et al. (2026). Briefly, leaf disks (19 mm diameter) were obtained from greenhouse-grown grapevine plants (*V. vinifera* cultivar Pinot Noir; from the third and fourth leaves from the apical meristem) with a cork borer, and they were placed randomly on four layers of wet filter paper in dishes(90 mm diameter; five leaf disks for each dish) with the abaxial surface uppermost. Leaf discs were sprayed with distilled water as control (CTR) or with 0.2 mM, 0.3 mM, 0.8 mM, 1.6 mM, 8 mM, or 16 mM CP using a compressed air hand sprayer (1 ml for each plate) and left to dry under a laminar flow hood for 10 min at room temperature. Leaf disks were inoculated with the P. viticola suspension (2.5 × 10^5^ sporangia/ml) using a hand sprayer (1 ml for each plate), incubated in the dark at 25 ± 1°C overnight, dried under a laminar flow hood chemical hood, and incubated for six days under greenhouse conditions (25 ± 1°C and 70 ± 10% RH) to allow downy mildew development. Downy mildew severity was assessed visually on each leaf disk at seven days post inoculation (dpi) as a percentage of the leaf disk surface covered by sporulation according to the guidelines of the European and Mediterranean Plant Protection Organization (EPPO) (EPPO 2001). The percentage of disease reduction (efficacy) was calculated for each leaf disc according to the following equation: (disease severity of CTR samples—disease severity of CP-treated samples) / disease severity of CTR samples × 100. Five biological replicates (dishes with leaf discs each) were assessed for each treatment.

### Assessment of choline pelargonate efficacy against downy mildew under greenhouse conditions

The efficacy against downy mildew under greenhouse conditions (efficacy trial) was assessed as described by Giovannini et al. (2022). Briefly, greenhouse-grown plants (*V. vinifera* cultivar Pinot Noir) were treated with distilled water as control (CTR) or with 1.6 mM, 8 mM, or 16 mM CP. A copper-based fungicide (Champ, Nufarm Italia, Milano, Italy) was used as reference fungicide (FUNG) at 2.5 g/l (corresponding to 0.9 mg/l copper ion), which is the label-recommended concentration and is consistent with standard viticultural practice against downy mildew (Dagostin et al. 2010). CP concentrations were chosen according to preliminary efficacy trials on leaf discs, and dosages with disease reduction (efficacy) > 90% were selected for the experiments under greenhouse conditions (Supplementary Table S1). All leaves of each plant were treated (30 ml per plant) to both surfaces (adaxial surface and abaxial surface; whole leaf treatment) one day before pathogen inoculation using an air compressor system (Advance, Fini, Bologna, Italy) equipped with an air spray gun working at 400 kPa pressure at the nozzle (Dagostin et al. 2010).

To assess the translaminar effect of CP against downy mildew (translaminar trial), greenhouse-grown grapevine plants were treated with distilled water (control; CTR), 16 mM CP, or 2.5 g/l copper (Champ, Nufarm Italia; corresponding to 0.9 mg/l copper ion) as reference fungicide (FUNG) to both surfaces of each leaf (whole leaf treatment) or only to the adaxial surface of each leaf (adaxial surface treatment). Treatments were applied one day before pathogen inoculation using a compressed air hand sprayer (15–30 ml for each plant).

In efficacy and translaminar trials, the abaxial surfaces of all leaves of each plant were inoculated with the *P. viticola* suspension (2.5 × 10^5^ sporangia/ml) one day after treatment, using an air compressor system (Advance, Fini, Turin, Italy) working at 400 kPa pressure (15–30 ml for each plant). Plants were incubated overnight in the dark at 25 ± 1°C with 97 ± 3% RH and maintained under greenhouse conditions (25 ± 1°C and 70 ± 10% RH) to allow pathogen infection and symptoms development. Six days after inoculation, plants were incubated overnight in the dark at 25 ± 1°C with 97 ± 3% RH to promote pathogen sporulation. The disease severity was visually assessed as the percentage of abaxial leaf area covered by white sporulation according to the reference scale reported by the EPPO guidelines (EPPO 2001). Six biological replicates (plants) were analyzed for each treatment in efficacy and translaminar trials, and each trial was carried out twice.

To assess the modulation of defense-related genes, leaf samples were collected from CTR and CP-treated (8 mM or 16 mM) plants of the efficacy trials just before *P. viticola* inoculation (T0), or one day (T1) and six days (T6) after *P. viticola* inoculation. Each sample comprised three half-leaves collected from the fourth to sixth node of each plant, as previously described (Avesani et al. 2023), and three biological replicates (plants) were analyzed for each treatment, time point, and experiment. Leaf samples were immediately frozen in liquid nitrogen and ground using a mixer-mill disruptor (MM 200, Retsch, Haan, Germany) with refrigerated stainless jars at 25 Hz for 30 sec, and the resulting powder was stored at −80°C until RNA extraction.

### Expression analysis of defense-related genes in grapevine leaves under greenhouse conditions

Total RNA was extracted from 80 mg of leaf powder using the Spectrum Plant Total RNA kit (Sigma–Aldrich, Merck, Rahway, NJ, USA) with an on-column DNase treatment with the RNase-Free DNase Set (Qiagen, Hilden, Germany). RNA was quantified by Qubit RNA Broad Range Assay Kit (Thermo Fisher Scientific, Waltham, MA, USA), and the effectiveness of the DNase treatment was confirmed by running PCR with grapevine *actin* primers (Supplementary Table S2) in the absence of reverse transcription, and no amplification signals were detected. The first strand cDNA was synthesized from 1.0 μg of total RNA using Superscript III (Invitrogen, Thermo Fisher Scientific) and oligo-dT primer. Genes encoding chitinase 3 (*CHIT-3*), lipoxygenase 9 (*LOX-9*), osmotin 2 (*OSM-2*), pathogenesis-related (PR) protein 1 (*PR-1*), protein 2 (*PR-2*), and protein 4 (*PR-4*) were used as markers of induced resistance mechanisms against downy mildew (Perazzolli et al. 2012). In particular, *PR-1* and *PR-2* are known as markers of the salicylic acid defense pathways (Gauthier et al. 2014), while *PR-4* and *LOX-9* are markers of the jasmonic acid defense pathways (Hamiduzzaman et al. 2005). The hypersensitive response-related gene (*HSR*) was used as a marker of cell death (Lakkis et al. 2019), while the stilbene synthase gene (*STS*) was used as a marker of the phenylpropanoid pathway (Lakkis et al. 2019).

Quantitative real-time PCR (qPCR) reactions were carried out with Platinum SYBR Green qPCR SuperMix-UDG (Invitrogen, Thermo Fisher Scientific) and specific primers (Supplementary Table S2) using the Light Cycler 480 (Roche, Thermo Fisher Scientific), as previously described (Avesani et al. 2023). Briefly, the PCR conditions were as follows: 50°C for 2 min and 95°C for 2 min as initial steps, followed by 40 cycles at 95°C for 15 s and at 60°C for 1 min. Each sample was examined in three technical replicates, and dissociation curves were analyzed to verify the specificity of each amplification reaction. The Light Cycler 480 SV 1.5.0 software (Roche, Thermo Fisher Scientific) was used to extract Ct values based on the second derivative calculation, and the reaction efficiency (Eff) was calculated with the LinRegPCR 11.1 software for each gene (Ruijter et al. 2009). The expression level of each gene was calculated according to the Hellemans equation (Hellemans et al. 2007), using *actin* and *VATP16* as housekeeping genes for normalization (Avesani et al. 2023). Briefly, relative quantities (RQ) were calculated according to the equation: RQ = Eff^(Ct–Ct′)^, where Ct is the threshold cycle and Ct’ is the mean Ct of CTR plants collected at T0. Normalized relative quantities (NRQ) were then calculated by dividing the RQ by the normalization factor based on the RQ values of the two housekeeping genes (Hellemans et al. 2007).

### Assessment of inhibitory effects against *Plasmopara viticola* sporangia and zoospores *in vitro*

The inhibitory effects on the viability of *P. viticola* sporangia (empty sporangia with zoospore release) and zoospores (motile zoospores) were assessed as described by Krzyzaniak et al. (2018) with slight modifications. To determine the viability of sporangia, the suspension (5 ml) of *P. viticola* sporangia (1 × 10^6^ sporangia/ml) was incubated in distilled water, augmented or not (control, CTR) with 1.6 mM, 8 mM, or 16 mM CP for 1 h at room temperature under orbital shaking at 120 r/m to allow zoospore release. An aliquot (50 µl) of sporangia suspension was transferred to a glass slide and observed under a light microscope (Eclipse 80i, Nikon). For each replicate, 100 sporangia were randomly observed in each glass slide, and sporangia viability was expressed as the percentage of empty sporangia (Perazzolli et al. 2008). Six biological replicates (tubes) were analyzed for each treatment, and the experiment was carried out twice.

To determine the viability of zoospores, the suspension (5 ml) of *P. viticola* sporangia (1 × 10^6^ sporangia/ml) was incubated for 1 h at room temperature under orbital shaking at 120 r/m to allow zoospore release. The sporangia suspension (5 ml) was augmented or not (control, CTR) with 1.6 mM, 8 mM, or 16 mM CP and incubated under orbital shaking at 120 r/m for 5 min at room temperature. An aliquot (50 µl) of sporangia suspension was transferred to a glass slide, and a video of 30 s was recorded under a light microscope (Leica TCS SP8, Leica Microsystems, Wetzlar, Germany). For each replicate, 100 zoospores were observed, and zoospore viability was expressed as the percentage of motile zoospores. Six biological replicates (tubes) were analyzed for each treatment, and the experiment was carried out twice.

### Assessment of choline pelargonate efficacy against powdery mildew under greenhouse conditions

The efficacy against powdery mildew under greenhouse conditions was assessed as described by Nesler et al. (2015). Briefly, greenhouse-grown plants (*V. vinifera* cultivar Pinot Noir) were treated with distilled water as control (CTR), with 1.6 mM, 8 mM, or 16 mM CP, or with 5 g/l sulfur (Microthiol Disperss, UPL Italia, San Carlo di Cesena, Italy) as reference fungicide (FUNG), corresponding to the label-recommended concentration and in agreement with standard viticultural practice against powdery mildew.

CP concentrations were chosen according to preliminary trials (data not shown). All leaves of each plant were treated (30 ml per plant) to both surfaces (adaxial surface and abaxial surface) of each leaf (whole leaf treatment) one day before pathogen inoculation with an air compressor system (Advance, Fini) equipped with an air spray gun working at 400 kPa pressure at the nozzle (Dagostin et al. 2010). One day after treatment, all leaves of each plant were inoculated with the *E. necator* suspension (2.5 × 10^4^ conidia/ml) using a compressed air hand sprayer (15–30 ml for each plant). Plants were incubated at 25 ± 1°C with 97 ± 3% RH for 24 h and maintained under greenhouse conditions (25 ± 1°C and 70 ± 10% RH) to allow pathogen infection and symptoms development. Fourteen days after pathogen inoculation, disease severity was visually assessed as the percentage of adaxial leaf area covered by white sporulation according to the reference scale reported by the EPPO guidelines (EPPO 2002). Six biological replicates (plants) were analyzed for each treatment, and the experiment was carried out twice.

### Assessment of inhibitory effects against *Erysiphe necator* conidia on leaf disks

The effect of CP on the germination of *E. necator* conidia was assessed as described by Nesler et al. (2015) with slight modifications. Leaves were collected from the third to fourth node of greenhouse-grown grapevine plants (*V. vinifera* cultivar Pinot Noir), and they were surface disinfected by incubation in 1% hypochlorite for 10 min and rinsed three times in sterile water by incubation for 5 min under orbital shaking at 80 r/m. Leaf disks (19 mm diameter) were obtained with a cork borer and were placed randomly on four layers of wet sterilized filter paper in dishes (90 mm diameter; four leaf disks for each dish) with the adaxial surface uppermost. Leaf disks were sprayed with distilled water as control (CTR) or with 1.6 mM, 8 mM, or 16 mM CP using a compressed air hand sprayer, and left to dry under a laminar hood for 1 h. Leaf discs were inoculated by brushing *E. necator* conidia, as described above, and incubated at 25 ± 1°C for 48 h in closed dishes (97 ± 3% RH) to allow conidial germination. A drop of 20 µl distilled water and 10 µl cotton blue staining solution (Peries 1962) was applied to each leaf disk, conidia were gently scraped with a loop, and the suspension was transferred to a glass slide by pipetting. The percentage of alive conidia (germinated conidia) was assessed by counting under a light microscope (Eclipse 80i, Nikon), and conidia were scored as germinated when their germ tube length was greater than their lateral radius (Pertot et al. 2007). Twelve biological replicates (leaf disks) were assessed for each treatment by counting all conidia of each leaf disk, and the experiment was carried out twice.

### Assessment of choline pelargonate efficacy against downy mildew and powdery mildew under field conditions

Two experimental vineyards (*V. vinifera* cultivar Merlot) were used for field experiments and they were located in San Michele all’Adige (vineyard 1, 46°10′55.8″ N, 11°07′38.3″ E; Trentino Alto Adige, Italy; altitude: 230 m above sea level, topography: low hill; rootstock: SO4, training system: guyot, planting pattern (m): 2.00 × 0.85; planting year: 2014; irrigation: drip irrigation) and Concesio (vineyard 2, 45°35′49.9″ N, 10°11′37.9″ E; Brescia, Italy; altitude: 269 m above sea level, topography: low hill; rootstock: SO4, training system: guyot, planting pattern (m) 2.50 × 2.00; planting year: 2004; irrigation: no irrigation). Plants were left untreated as control (CTR), treated with 16 mM CP (corresponding to 10 ml/l of the stock solution at 41%) optimized in the greenhouse experiments, or with a combination of copper and sulfur as reference fungicides (FUNG) against downy mildew and powdery mildew in organic viticulture (Supplementary Table S3). Untreated plants were used as control, since water treatment did not cause major changes in the taxonomic structure of bacterial and fungal communities on grapevine leaves (Cappelletti et al. 2016). Field experiments were carried out in a randomized block design, and four plots, consisting of twelve plants each, were used as biological replicates for each treatment, as previously described (Giovannini et al. 2022). Meteorological data were recorded by stations near the two vineyards (46°11′24.1″N, 11°08′04.9″E and 45°38′58.7“N 10°12′18.3″E, respectively).

Plants were treated every 3–13 days before predicted rainfall and probable infection of *P. viticola*, according to the weather forecast (https://www.3bmeteo.com/) and DSS RIMpro-Plasmopara (https://rimpro.cloud/fr/platform/downy-mildew-plasmopara/), with a spray volume of 520-830 L/ha depending on the phenological stage (Supplementary Table S3), in agreement with standard viticultural practice applied against downy mildew in Northern Italy (Dagostin et al. 2010). In vineyard 1, copper (Bordoflow Sector, Manica, Rovereto, Italy) was applied at optimized rates throughout the season, depending on the phenological stage and weather conditions suitable for *P. viticola* infections, with a maximum concentration of 3.2 ml/l per treatment (corresponding to 393.3 g/ha copper ion) and a total amount of 2.7 kg/ha copper ion applied in the whole season (Supplementary Table S3). Sulfur (Microthiol Disperss, UPL Italia, San Carlo di Cesena, Italy) was applied in tank mixture with copper at a constant rate of 5 g/l in vineyard 1 (Supplementary Table S3). In vineyard 2, copper (Bordoflow Sector, Manica, Rovereto, Italy) and sulfur (Microthiol Disperss, UPL Italia, San Carlo di Cesena, Italy) were applied in tank mixture at a constant rate of 6.7 ml/l (corresponding to 498.5 g/ha copper ion) and 6.7 g/l, respectively, with a total amount of 4.5 kg/ha copper ion applied in the whole season (Supplementary Table S3). Two insecticide treatments (acetamiprid, 1.5 ml/l, Epik SL, Sipcam Italia, Milano, Italy; and etofenprox, 0.5 ml/l, Trebon up, Sipcam Italia, Milano, Italy) and one insecticide treatment (acetamiprid, 2.5 ml/L, Epik SL, Sipcam Italia) were applied to all plants in vineyard 1 and vineyard 2 against *Scaphoideus titanus*, respectively (Supplementary Table S3). Products were dissolved in tap water and sprayed with a Solo 450 motorized backpack mist blower (Solo, Newport News, VA, USA) in vineyard 1 and with an SR 430 motorized backpack mist blower (Stihl, Waiblingen, Baden-Württemberg, Germany) in vineyard 2.

Downy mildew development was evaluated constantly in the two vineyards, and disease severity was assessed visually starting from the first disease appearance as a percentage of abaxial leaf area or bunch area covered by downy mildew symptoms (oil spots, *P. viticola* sporulation, and/or tissue necrosis) on 60 leaves or 60 bunches per plot, according to reference scale reported by the EPPO guidelines (EPPO 2001). Disease assessments were carried at different phenological stages defined according to the scale of Biologische Bundesanstalt, Bundessortenamt und CHemical industry (BBCH) (Lorenz et al. 1995), such as three time points (5^th^ June 2024, BBCH-70; 20^th^ June 2024, BBCH-73; and 4^th^ July 2024, BBCH-77) on leaves and bunches in vineyard 1, six time points on leaves in vineyard 2 (4^th^ June 2024, BBCH-65; 14^th^ June 2024, BBCH-71, 20^th^ June, BBCH-73; 28^th^ June 2024 BBCH-75; 11^th^ July 2024, BBCH-77 and 19^th^ July 2024, BBCH-78) and three time points on bunches in vineyard 2 (28^th^ June 2024, BBCH-75; 11^th^ July 2024, BBCH-77 and 19^th^ July 2024, BBCH-78; Supplementary Table S3). Powdery mildew severity was assessed as percentage of adaxial leaf area or bunch area covered by powdery mildew symptoms (*E. necator* sporulation or tissue necrosis) on 60 leaves or 40 bunches per plot, according to reference scale reported by the EPPO guidelines (EPPO 2002), on 8^th^ July 2024 (BBCH-77) in vineyard 1 and 19^th^ July 2024 (BBCH-77) in vineyard 2 (Supplementary Table S3).

### Collection of phyllosphere micro-organisms under field conditions

Phyllosphere micro-organisms were collected by leaf and bunch washing as previously described (Perazzolli et al. 2014) with slight modifications. Leaves and bunches were randomly collected from control plants (CTR) and plants treated with CP or with a combination of copper and sulfur as reference fungicides (FUNG) at two time points from each vineyard: at the end of blooming (after six and four treatments in vineyard 1 and vineyard 2, respectively), namely T1 (2^nd^ June 2024, BBCH-69 in vineyard 1 and 3^rd^ June 2024, BBCH-63 in vineyard 2), and at bunch closure (after eleven and eight treatments in vineyard 1 and vineyard 2, respectively), namely T2 (1^st^ July 2024, BBCH-77 in vineyard 1 and 3^rd^ July 2024, BBCH-76 in vineyard 2), corresponding to the most critical phases for *P. viticola* and *E. necator* infections (Supplementary Table S3). For each time point, asymptomatic leaf and bunch samples were randomly collected from the four plots for each treatment and vineyard, and five biological replicates (pool of 20 leaves or 12 bunches each) were obtained and transported to the laboratory in a cool box, stored in a refrigerator at 4°C, and processed within 24 h after collection. Each leaf or bunch sample was transferred into a sterile plastic box containing 750 ml or 250 ml of washing solution [0.85% (w/v) NaCl and 0.01% (v/v) Tween 80], respectively, and incubated under orbital shaking for 15 min at 120 r/m. The suspension was filtered with a sterile gauze, centrifuged at 10 000 × g for 20 min at 4°C, and the resulting pellets were stored at −20°C until DNA extraction.

### DNA extraction, amplification, and sequencing of phyllosphere micro-organisms

DNA extraction, amplification, and sequencing of leaf- and bunch-associated micro-organisms were carried out as previously described (Perazzolli et al. 2020). The genomic DNA was extracted using the FastDNA spin kit for soil (MP Biomedicals, Irvine, CA, USA) and quantified with a Qubit DNA High Sensitivity Assay Kit (Invitrogen, Thermo Fisher Scientific). The bacterial V5-V7 region of 16S ribosomal RNA (rRNA) gene and the fungal ITS2 region of the internal transcribed spacer (ITS) were amplified from 5 ng template DNA with a nested PCR approach, as previously described (Perazzolli et al. 2022), to limit the amplification of host DNA in amplicon sequencing studies of plant endophytes (Mitter et al. 2017, Escobar Rodríguez et al. 2018, Keiblinger et al. 2018, Stefani et al. 2020, Buchholz et al. 2021). The first bacterial 16S amplification was carried out with the primer 799 forward (5′-AACMGGATTAGATACCCKG-3′) and 1392 reverse (5′-ACGGGCGGTGTGTRC-3′), to exclude chloroplast 16S rRNA gene and to amplify bacterial and mitochondrial rRNA gene of 600 bp and 1000 bp amplicon size, respectively (Mitter et al. 2017). The first fungal ITS amplification was carried out with the primers ITS1 forward (5′-CTTGGTCATTTAGAGGAAGTAA-3′) and TW13 reverse (5′-GGTCCGTGTTTCAAGACG-3′), which amplify fungal ITS and part of the ribosomal large subunit (Klaubauf et al. 2010). Bacterial amplicons (600 bp) and fungal amplicons (500–600 bp) were purified by agarose gel separation, followed by the NucleoSpin Gel and PCR Clean-up purification kit (Macherey-Nagel, Düren, Germany). The second 16S amplification was carried out using the purified product (2 µl) with the primer 799 forward (5′-AACMGGATTAGATACCCKG-3′) and 1175 reverse (5′-ACGTCRTCCCCDCCTTCCT-3′) (Perazzolli et al. 2022). The second ITS amplification was carried out using the purified product (2 µl) with equimolar mixes of the degenerated ITS3 forward primer (5′-CAHCGATGAAGAACGYRG-3′) (Tedersoo et al. 2014) and the degenerated ITS4 reverse primer (5′-TCCTSSSCTTATTGATATGC-3′) (Keiblinger et al. 2018, Perazzolli et al. 2022). Primers of the second 16S and ITS amplification included the specific overhang Illumina adapters (5′-ACACTCTTTCCCTACACGACGCTCTTCCGATCT-3′ and 5′-GACTGGAGTTCAGACGTGTGCTCTTCCGATCT-3′ for forward and reverse primers, respectively) for the amplicon library construction. Bacterial 16S and fungal ITS amplifications were obtained using the FastStart High-Fidelity PCR system (Roche, Thermo Fisher Scientific) with PCR conditions optimized for each amplification (Supplementary Table S4).

PCR product purification, quantification, library construction, and sequencing were conducted by Eurofins Genomics GmbH (Ebersberg, Germany) following the NGSelect Amplicons approach with an index PCR to introduce indexed sequencing adaptors and Illumina MiSeq (PE300 mode) sequencing. Bacterial (16S) and fungal (ITS) sequences were obtained from two vineyards (vineyard 1 and vineyard 2), two time points (T1 and T2), two plant compartments (leaves and bunches), three treatments (CTR, CP, and FUNG) and five biological replicates, for a total of 120 samples.

### Bioinformatic analysis and sequence processing

Bioinformatic analysis of amplicon sequencing data was carried out as described by Jaeger et al. (2023) with slight modifications. Sequencing data were analyzed using a customized bioinformatics pipeline implemented through Bash scripting and based on VSEARCH v2.21.1 (Rognes et al. 2016), and PCR primers were trimmed using CUTADAPT v3.4, allowing for two mismatches (Martin 2011). PhiX contamination was filtered out using Bowtie2 v2.4.5 (Langmead and Salzberg 2012) by aligning reads against the PhiX genome (accession NC_001422.1). Trimmed paired-end reads were merged using the *fastq_mergepairs* function of VSEARCH v2.21.1, and quality filtering was performed using the *fastq_filter* function, allowing a maximum expected error of one (Edgar and Flyvbjerg 2015). Filtered reads were dereplicated using the *derepfulllength* function of VSEARCH v2.21.1 and delineated into amplicon sequence variants (ASVs) using the UNOISE algorithm (Edgar 2016c) implemented within VSEARCH v2.21.1 with an *alpha* of two and a *minsize* of eight. Potential chimeric sequences were removed using the UCHIME2 algorithm (Edgar 2016b), incorporated as the *uchime3_denovo* function of VSEARCH v2.21.1. The remaining ASV sequences were screened for ribosomal gene signatures using Metaxa2 v.2.2.3 for the 16S rRNA regions (Bengtsson-Palme et al. 2015) and ITSx v.1.1.3 for ITS2 regions (Bengtsson-Palme et al. 2013), and sequences without matching signatures were discarded. The final ASV table was generated by mapping the filtered reads of each sample against the ASV sequences using the *usearch_global* algorithm implemented in VSEARCH v2.21.1, with parameters set as follows: *maxhits* of one, *maxrejects* of 50, *maxaccepts* of 50, and a minimum sequence identity threshold of 97%. ASVs were taxonomically annotated using the SINTAX algorithm (Edgar 2016a), implemented in VSEARCH v2.21.1, against the SILVA v.138 database (Quast et al. 2012) for 16S rRNA gene sequences, and against the UNITE v.10.0 database (Abarenkov et al. 2010) for ITS2 sequences, applying a bootstrap threshold of 0.8. ASVs not assigned to bacterial, archaeal, or fungal taxa, and sequences classified as chloroplast or mitochondrial sequences were filtered out from the ASV table. No reads of oomycetes were found, possibly due to the absence of downy mildew sporulation on collected samples, the washing protocol not being able to successfully collect *P. viticola* hyphae, and/ or the primers not being suitable to amplify oomycetes.

### Statistical analysis

Statistical analyses were carried out in R, using RStudio Version 2022.02.1 + 461. For disease severity and spore vitality data, the Kruskal–Wallis test was used to demonstrate non-significant (*P* > 0.05) experiment interaction (comparable results among experimental repetitions) using the *kruskal_test* function from the *rstatix* package v.0.7.2. Normal distribution (Shapiro test, *P* > 0.05) and variance homogeneity (Levene test, *P* > 0.05) of the data were checked using the *shapiro.test* function from the *stat* package v.3.6.2 and the *leveneTest* function from the *car* package v.3.1–3. Significant differences among treatments were assessed with the Kruskal–Wallis test (*P* ≤ 0.05) followed by the Dunn’s test (*P* ≤ 0.05) using the *dunn.test* function from the *dunn.test* package v1.3.6 when parametric assumptions were not satisfied. Values of the gene expression analysis were Log_10_-transformed (Lazazzara et al. 2021), and a Student’s t-test was used to detect significant differences between CP-treated and CTR plants for each time point (*P* ≤ 0.05) using *t.test* function from the package *stats* v.3.6.2.

Analyses of amplicon sequencing data showed major effects of the plant compartment on taxonomic structure of bacterial and fungal communities (Supplementary Table S5), and the dataset was split into leaf communities and bunch communities to better highlight the effects of vineyard, time point, and treatments. Rarefaction curves were generated with the *rarecurve* function from the *vegan* package v.2.6–2 in R (Oksanen et al. 2013). ASVs tables were iteratively subsampled (100 times) using the *rrarefy* function from the *vegan* package v.2.6–2 (Martiny et al. 2017, Schloss Patrick 2024), according to the minimum sequencing depth of each dataset (corresponding to 16 156 reads for leaf bacteria, 65 109 reads for bunch bacteria, 56 272 reads for leaf fungi, and 56 218 reads for bunch fungi). Alpha-diversity metrics (number of observed ASVs, Pielou’s evenness index, and Shannon index) and beta-diversity metrics (Bray–Curtis dissimilarity matrices) were calculated from the subsampled ASV tables, applying the *specnumber, diversity* and *vegdist* functions of the *vegan* package v.2.6–2, respectively (Martiny et al. 2017, Hemkemeyer et al. 2019). The effects of vineyard, time point, and treatment on alpha-diversity and beta-diversity metrics were evaluated with univariate and multivariate permutational analysis of variance (PERMANOVA) using the *adonis2* function of the *vegan* package v.2.6–2 with 999 permutations, respectively (Anderson 2001). Moreover, the homogeneity of group dispersion on alpha-diversity and beta-diversity metrics was evaluated with permutational analysis of multivariate dispersions (PERMDISP) using the *betadisper* function of the *vegan* package v.2.6- 2 with 999 permutations (Anderson 2005). Comparisons of alpha-diversity metrics among vineyards (vineyard 1 and vineyard 2), time points (T1 and T2), and treatments (CTR, CP, and FUNG) were carried out with pairwise PERMANOVA using the *pairwise.adonis2* function of the pairwiseAdonis v0.4 package with 999 permutations (Arbizu 2020). Pairwise PERMANOVA with 999 permutations (*pairwise.adonis2* function of the pairwiseAdonis v0.4 package) (Arbizu 2020) was applied on beta-diversity metrics to detect significant differences among treatments (CTR *vs*. CP, CTR *vs*. FUNG, and FUNG *vs*. CP). Moreover, beta-diversity was evaluated by unconstrained ordination using principal coordinate analysis (PCoA) implemented with the *cmdscale* function (Gower 2014), and constrained ordination using canonical analysis of principal coordinates (CAP) (Anderson and Willis 2003) implemented with the *CAPdiscrim* function of the *BiodiversityR* package v.2.14–1 using the Bray-Curtis dissimilarity matrices with 999 permutations (Kindt 2020). Vineyard, time point, and treatment were used as constraining factors, and the reclassification success, expressed as a percentage of correctly assigned samples, was used to assess the accuracy of the CAP model in grouping samples (Jaeger et al. 2024).

Read counts of each ASV assigned to the same genus were aggregated, and the effects of vineyard, time point, and treatment on the relative abundances of bacterial and fungal genera were evaluated with multivariate PERMANOVA (*adonis2* function) and PERMIDSP (*betadisper* function) using the *vegan* package v.2.6–2 with Bray–Curtis dissimilarity matrices and 999 permutations. To select bacterial and fungal genera whose relative abundance was significantly affected by treatments (responsive genera; *q*-value ≤ 0.05), an univariate PERMANOVA was performed on the relative abundance data of each genus (*adonis2* function with 999 permutations), and *q*-values were calculated using the *qvalue* v.2.24.0 package (Storey and Tibshirani 2003). For each responsive genus, pairwise comparisons were carried out to identify significant changes in relative abundances between CTR, CP-treated, and FUNG-treated samples using a pairwise PERMANOVA with 999 permutations (Jaeger et al. 2024). Relative abundance data were Z-transformed using the *scale* function of R (i.e. for each genus, the mean relative abundance across all samples was subtracted from each relative abundance value, and the result was divided by the standard deviation across all samples) to standardize differences across genera (Krause et al. 2025), and relative changes in abundance were calculated as the difference of Z-transformed relative abundances between CP-treated and CTR samples or FUNG-treated and CTR samples for each responsive genus. The interaction between treatment and time point was also evaluated to highlight genera with different responses to the treatments at T1 and T2 (*q*-value ≤ 0.05).

## Results

### Choline pelargonate efficacy against downy mildew and powdery mildew under controlled conditions

**Figure 1:**
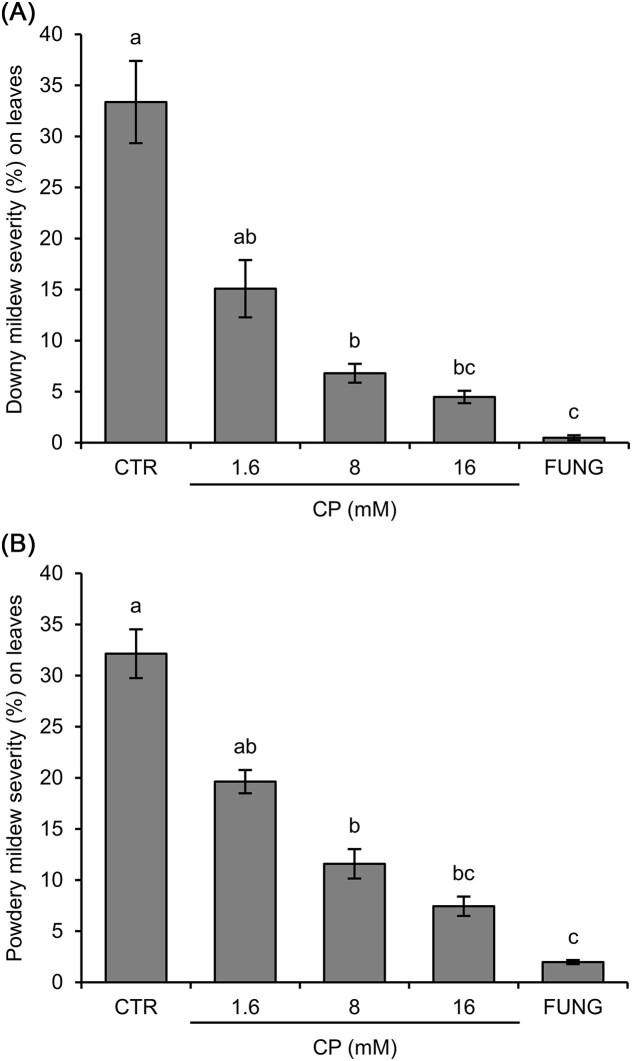
Choline pelargonate decreased downy mildew and powdery mildew severity under greenhouse conditions. Plants were treated to both surfaces of each leaf (whole leaf treatment) with water (control; CTR), 1.6 mM, 8 mM, or 16 mM choline pelargonate (CP), while copper and sulfur were applied as reference fungicides (FUNG) against downy mildew (A) and powdery mildew (B), respectively. Downy mildew severity and powdery mildew severity were assessed as the percentage of leaf area with disease symptoms at seven and 14 days post inoculation (dpi), respectively. Comparable results were obtained between the two experiments (Kruskal–Wallis *P* > 0.05), and data were pooled. Mean and standard error values of 12 biological replicates (plants) from two experiments are reported for each treatment. Different letters indicate significant differences among treatments, according to the Kruskal–Wallis test followed by the Dunn’s test (*P* ≤ 0.05).

CP treatment decreased downy mildew severity under greenhouse conditions, and the disease reduction (efficacy) was 79.6 ± 2.3% and 86.6 ± 2.1% (mean ± standard error) for CP concentrations of 8 mM and 16 mM, respectively (Fig. 1A). In particular, downy mildew severity of plants treated with 16 mM CP was comparable to that of plants treated with copper (FUNG; *P* > 0.05 of the Kruskal–Wallis test followed by the Dunn’s test), and this CP concentration was selected for the field experiments. CP treatment upregulated the expression of the grapevine defense-related genes *CHIT-3* and *OSM-2* before *P. viticola* inoculation (8 mM and 16 mM CP) and the expression of *PR-1* (marker of the salicylic acid pathway) one day after *P. viticola* inoculation (16 mM CP; Supplementary Fig. S1). Moreover, the *P. viticola*-dependent upregulation of *HSR* (marker of hypersensitive response), *LOX-9* (marker of the jasmonic acid pathway), *PR-2* (marker of the salicylic acid pathway), and *STS* (marker of the phenylpropanoid pathway) was decreased in CP-treated compared to control plants at six days after *P. viticola* inoculation, possibly due to the lower *P. viticola* infection level (Supplementary Fig. S1). CP decreased the viability of *P. viticola* sporangia and zoospores, with comparable inhibitory effects at 1.6 mM, 8 mM, and 16 mM concentrations (Supplementary Fig. S2A and S2B). Downy mildew severity was comparable between CP-treated and control plants in adaxial leaf treatments, indicating no translaminar effects of CP (Supplementary Fig. S3).

CP treatment decreased powdery mildew severity under greenhouse conditions, and the disease reduction was comparable at 8 mM (63.9 ± 4.5%) and 16 mM (76.8 ± 2.9%; Fig. 1B). Moreover, powdery mildew severity was comparable in CP-treated (16 mM concentration) and sulfur-treated plants (FUNG; *P* > 0.05 of the Kruskal–Wallis test followed by the Dunn’s test), and this CP concentration was selected for the field experiments. CP inhibited the germination of *E. necator* conidia on leaf disks in a dose-dependent manner (Supplementary Fig. S2C).

### Choline pelargonate efficacy against downy mildew and powdery mildew under field conditions

**Figure 2:**
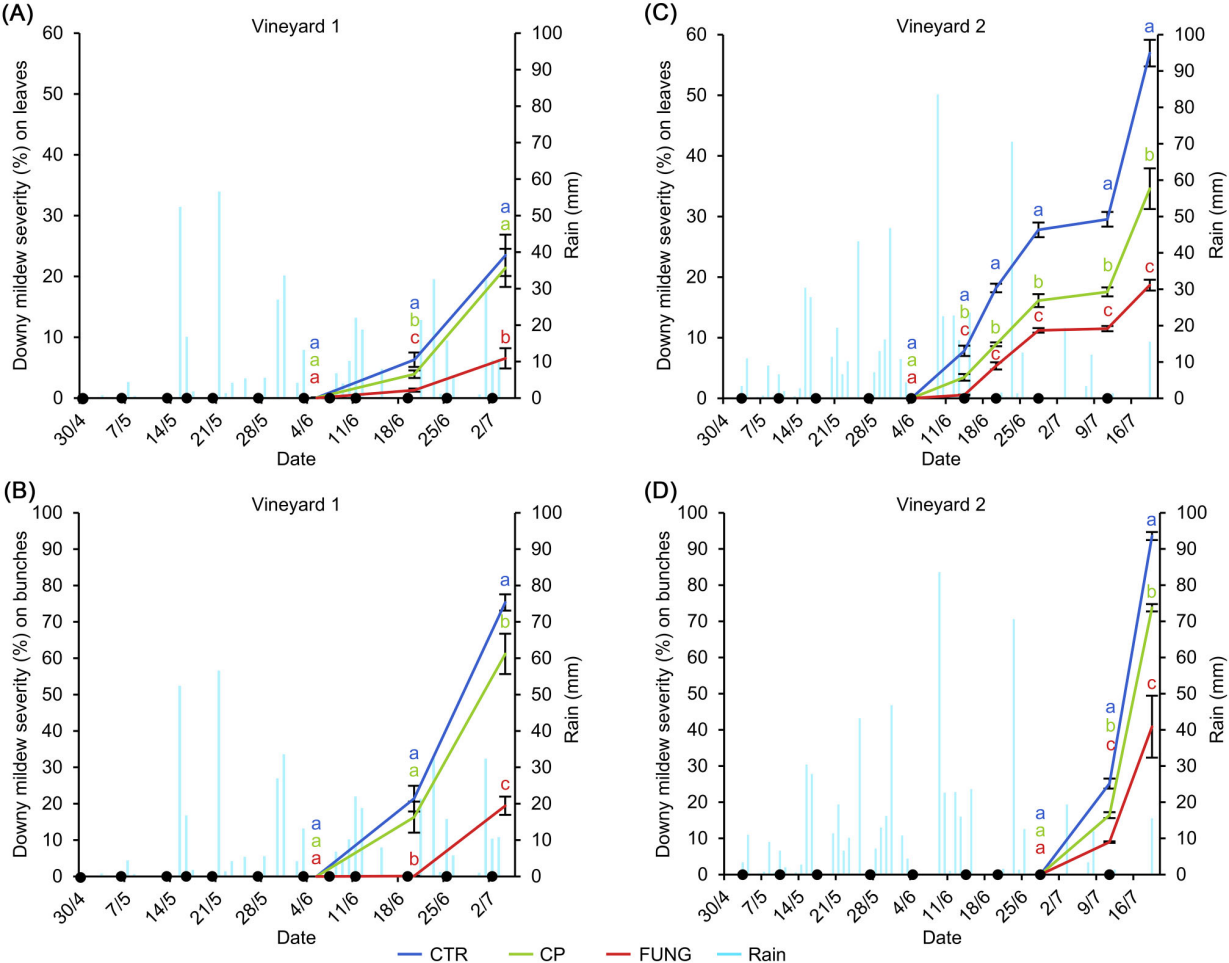
Choline pelargonate decreased downy mildew severity under field conditions. Downy mildew severity was assessed on leaves and bunches of control plants (CTR; blue lines) and plants treated with 16 mM choline pelargonate (CP; green lines) or with a combination of copper and sulfur as reference fungicides (FUNG; red lines) in vineyard 1 (A, B) and vineyard 2 (C, D). The rainfall (mm) is indicated by the vertical cyan bars. Twelve and nine treatments (black dots) were applied to vineyard 1 and vineyard 2, respectively. Four plots of twelve plants each were analyzed for each treatment in a randomized block design, and downy mildew severity was assessed as the percentage (%) of leaf area or bunch area with downy mildew symptoms on 60 leaves or 60 bunches per plot. Disease severity assessments were carried out starting from the first disease appearance at three time points (5^th^ June, BBCH-70; 20^th^ June, BBCH-73; and 4^th^ July, BBCH-77) on leaves and bunches in vineyard 1, and at six time points on leaves (4^th^ June, BBCH-65; 14^th^ June, BBCH-71, 20^th^ June, BBCH-73; 28^th^ June, BBCH-75; 11^th^ July, BBCH-77 and 19^th^ July, BBCH-78) and three time points on bunches (28^th^ June, BBCH-75; 11^th^ July, BBCH-77 and 19^th^ July, BBCH-78) in vineyard 2. Mean and standard error values of four biological replicates (plots) are reported for each time point and treatment. For each assessment, different letters indicate significant differences among treatments, according to the Kruskal-Wallis test followed by the Dunn’s test (*P* ≤ 0.05).

Weather conditions were favorable to *P. viticola* infections, and the disease severity was 23.5 ± 3.4% on leaves and 75.3 ± 2.2% on bunches of control plants in vineyard 1, while it was 57.0 ± 2.2% on leaves and 93.6 ± 1.1% on bunches of control plants vineyard 2 at the last assessment (BBCH-77: 4^th^ July 2024 in vineyard 1 and 19^th^ July 2024 in vineyard 2; Fig. 2 and Supplementary Fig. S4). In vineyard 1, 12 treatments were applied (Supplementary Table S3), and downy mildew severity on leaves and bunches was lower in CP-treated compared to control plants at BBCH-73 (20^th^ June 2024; Fig. 2A) and BBCH-77 (4^th^ July 2024; Fig. 2B), respectively. In vineyard 2, nine treatments were carried out (Supplementary Table S3), and downy mildew severity on leaves and bunches was lower in CP-treated compared to control plants at five time points (BBCH-71, 14^th^ June; BBCH-73, 20^th^ June; BBCH-75, 28^th^ June; BBCH-77, 11^th^ July; and BBCH-77, 19^th^ July; Fig. 2C) and two time points (BBCH-77, 11^th^ July and BBCH-77, 19^th^ July; Fig. 2D), respectively. Downy mildew severity on leaves and bunches was higher in CP-treated plants compared to plants treated with the combination of copper and sulfur as reference fungicides for organic viticulture (FUNG) at all time points in both vineyards.

**Figure 3:**
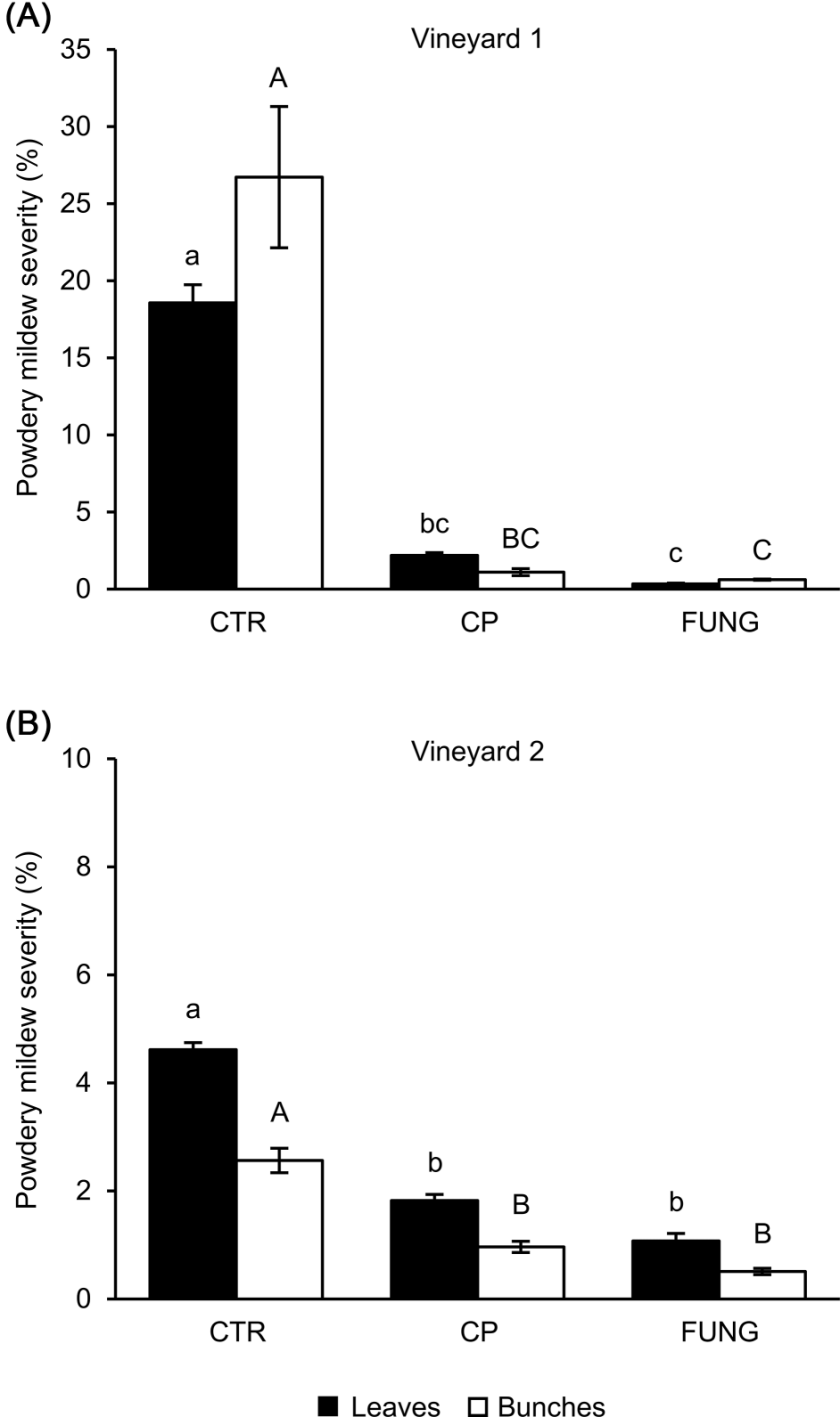
Choline pelargonate decreased powdery mildew severity under field conditions. Powdery mildew severity was assessed on leaves (black) and bunches (white) of control plants (CTR) and plants treated with 16 mM choline pelargonate (CP) or with a combination of copper and sulfur as reference fungicides (FUNG) in vineyard 1 (A) and vineyard 2 (B). Four plots of twelve plants were analyzed for each treatment in a randomized block design, and powdery mildew severity was assessed as percentage (%) of adaxial leaf area or bunch area covered by powdery mildew symptoms on 60 leaves or 40 bunches per plot at BBCH-77 (8^th^ July in vineyard 1 and 19^th^ July in vineyard 2). Mean and standard error values of four biological replicates (plots) are reported for each treatment. Lowercase and uppercase letters indicate significant differences among treatments for leaves and bunches, respectively, according to the Kruskal-Wallis test followed by the Dunn’s test (*P* ≤ 0.05), respectively.

Weather conditions were favorable to powdery mildew infections, and the disease severity was 18.6 ± 1.2 on leaves and 26.7 ± 4.6% on bunches of control plants in vineyard 1, while it was 4.6 ± 2.6% on leaves and 2.6 ± 0.2% on bunches of control plants in vineyard 2 at BBCH-77 (Fig. 3). In both vineyards, powdery mildew severity on leaves and bunches was lower in CP-treated compared to control plants, and it was comparable in CP-treated plants and plants treated with the combination of copper and sulfur as reference fungicides (FUNG).

### Effects of vineyard, time point, and treatment on the taxonomic structure of bacterial and fungal communities of grapevine leaves

After quality filtering and taxonomic assignment, 9 948 118 16S rRNA gene and 9 223 674 ITS sequences were obtained from grapevine leaf and bunch samples collected at two time points (T1, blooming and T2, bunch closure) in vineyard 1 and vineyard 2 (Supplementary Fig. S5 and Table S6), and they were delineated into 8777 bacterial ASVs and 2000 fungal ASVs (Supplementary Tables S7 and S8). Major effects of the plant compartment were found on the taxonomic structure of bacterial communities (PERMANOVA; F = 20.48, *P* = 0.001) and fungal communities (F = 54.82, *P* = 0.001; Supplementary Table S5), and datasets of leaf communities and bunch communities were analyzed separately.

**Figure 4:**
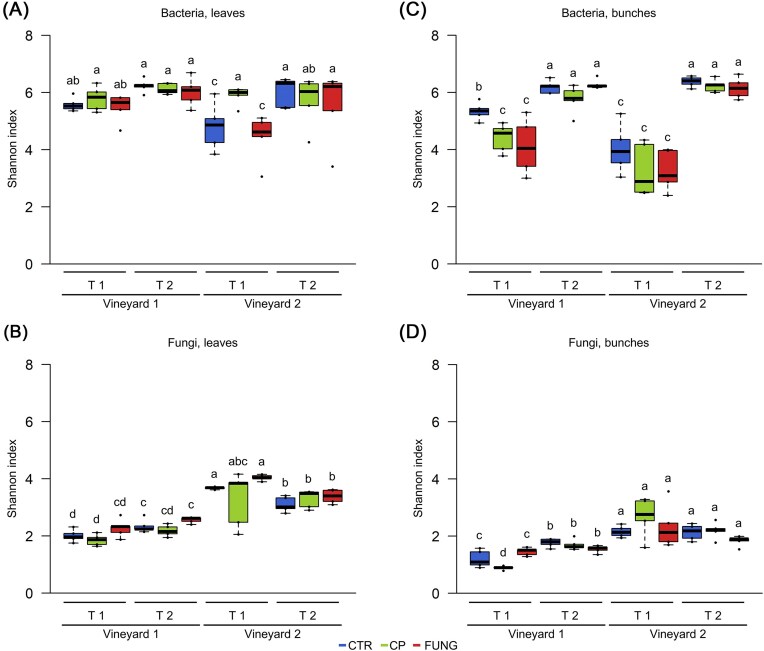
Alpha-diversity (Shannon index) of grapevine phyllosphere micro-organisms was affected by vineyard, time point, and/or treatment. The Shannon index of bacterial and fungal communities was assessed on leaves (A, B) and bunches (C, D) of control plants (CTR; blue) and plants treated with 16 mM choline pelargonate (CP; green) or with a combination of copper and sulfur as reference fungicides (FUNG; red), collected at blooming (T1) and bunch closure (T2) in vineyard 1 and vineyard 2. The effects of vineyard, time point, and treatment were evaluated using univariate permutational analysis of variance (PERMANOVA with 999 permutations; Supplementary Table S9). Different letters indicate significant differences among samples, according to pairwise PERMANOVA (999 permutations; *P ≤* 0.05).

Time point and vineyard were the main drivers of bacterial alpha-diversity of grapevine leaves, such as Shannon index (F = 14.09, *P* = 0.001, and F = 8.30, *P* = 0.004, respectively), number of observed ASVs (F = 36.67, *P* = 0.001, and F = 6.71, *P* = 0.016, respectively), and Pielou’s evenness index (F = 7.88, *P* = 0.001, and F = 6.95, *P* = 0.007, respectively; Supplementary Table S9A). Moreover, bacterial alpha-diversity metrics of grapevine leaves tended to be lower for CTR and FUNG-treated samples of vineyard 2 at T1 compared to the other samples (Fig. 4A, Supplementary Figs. S6A and S7A). Fungal alpha-diversity metrics of grapevine leaves were mainly affected by the vineyard, such as Shannon index (F = 202.98, *P* = 0.001), number of observed ASVs (F = 525.57, *P* = 0.001), and Pielou’s evenness index (F = 149.86, *P* = 0.001; Supplementary Table S9B), and they were higher in vineyard 2 compared to vineyard 1 (Fig. 4B, Supplementary Figs. S6B and S7B).

**Figure 5:**
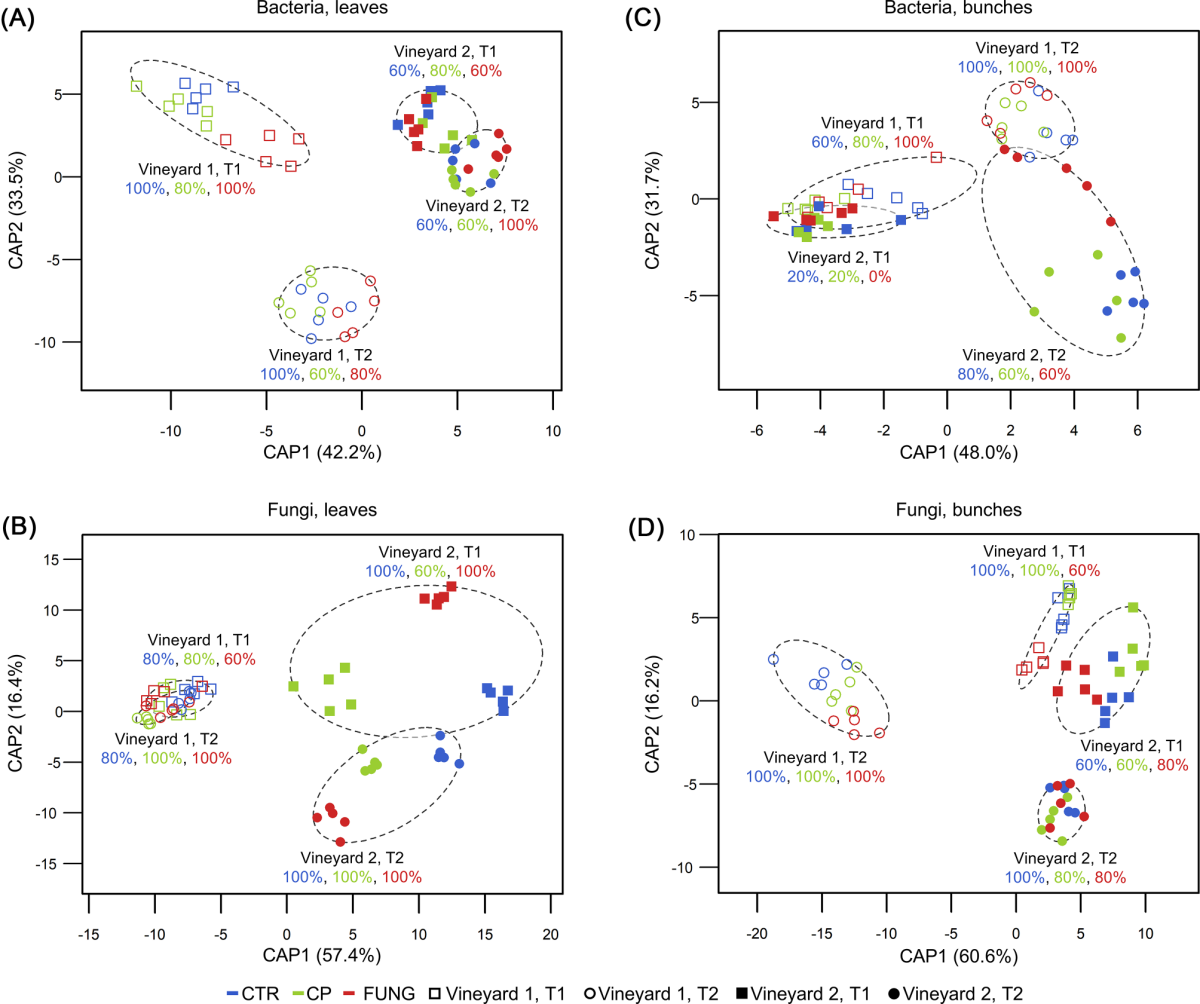
Constrained analysis of principal coordinates (CAP) of grapevine phyllosphere micro-organisms. CAP was obtained for bacterial and fungal communities of leaves (A, B) and bunches (C, D) collected from control plants (CTR; blue) and plants treated with 16 mM choline pelargonate (CP; green) or with a combination of copper and sulfur as reference fungicides (FUNG; red), at blooming (T1; square symbols) and bunch closure (T2; circle symbols) in vineyard 1 (empty symbols) and vineyard 2 (solid symbols) based on Bray–Curtis dissimilarity matrices. The effects of vineyard, time point, and treatment were evaluated using multivariate permutational analysis of variance (PERMANOVA with 999 permutations; Supplementary Table S10). Dashed ellipses enclose samples from the same vineyard and time point, and percentages indicate the reclassification success of samples (percentage of correctly assigned samples).

Beta-diversity analysis of leaf samples showed that bacterial communities (Fig. 5A and Supplementary Fig. S8A) and fungal communities (Fig. 5B and Supplementary Fig. S8B) clustered according to vineyard and time point, with a mean reclassification success of 82.5%. Vineyard and time point were the main drivers of beta-diversity for bacterial communities (F = 10.39, *P* = 0.001 and F = 7.53, *P* = 0.001, respectively; Supplementary Table S10A) and fungal communities (F = 92.42, *P* = 0.001 and F = 14.88, *P* = 0.001, respectively; Supplementary Table S10B) on grapevine leaves. Moreover, the treatment affected the beta-diversity of bacterial communities (F = 2.80, *P* = 0.001; Supplementary Table S10A) and fungal communities (F = 5.46, *P* = 0.001; Supplementary Table S10B), and bacterial communities on grapevine leaves differed in FUNG-treated samples compared to CTR and CP-treated samples (Supplementary Table S10A).

**Figure 6.**
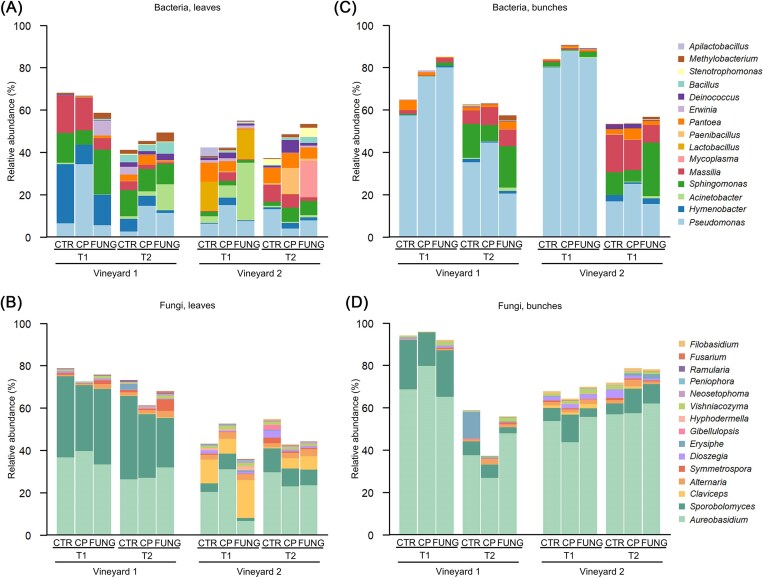
The vineyard, time point, and treatment influenced the relative abundances of dominant genera. Relative abundances of the fifteen most abundant bacterial and fungal genera (dominant genera, relative abundance > 4% for bacterial genera and > 0.6% for fungal genera) were assessed on leaves (A, B) and bunches (C, D) of control plants (CTR) and plants treated with 16 mM choline pelargonate (CP) or with a combination of copper and sulfur as reference fungicides (FUNG), collected at blooming (T1) and bunch closure (T2) in vineyard 1 and vineyard 2. The effects of vineyard, time point, and treatment were evaluated using multivariate permutational analysis of variance (PERMANOVA with 999 permutations; Supplementary Table S13).

**Figure 7:**
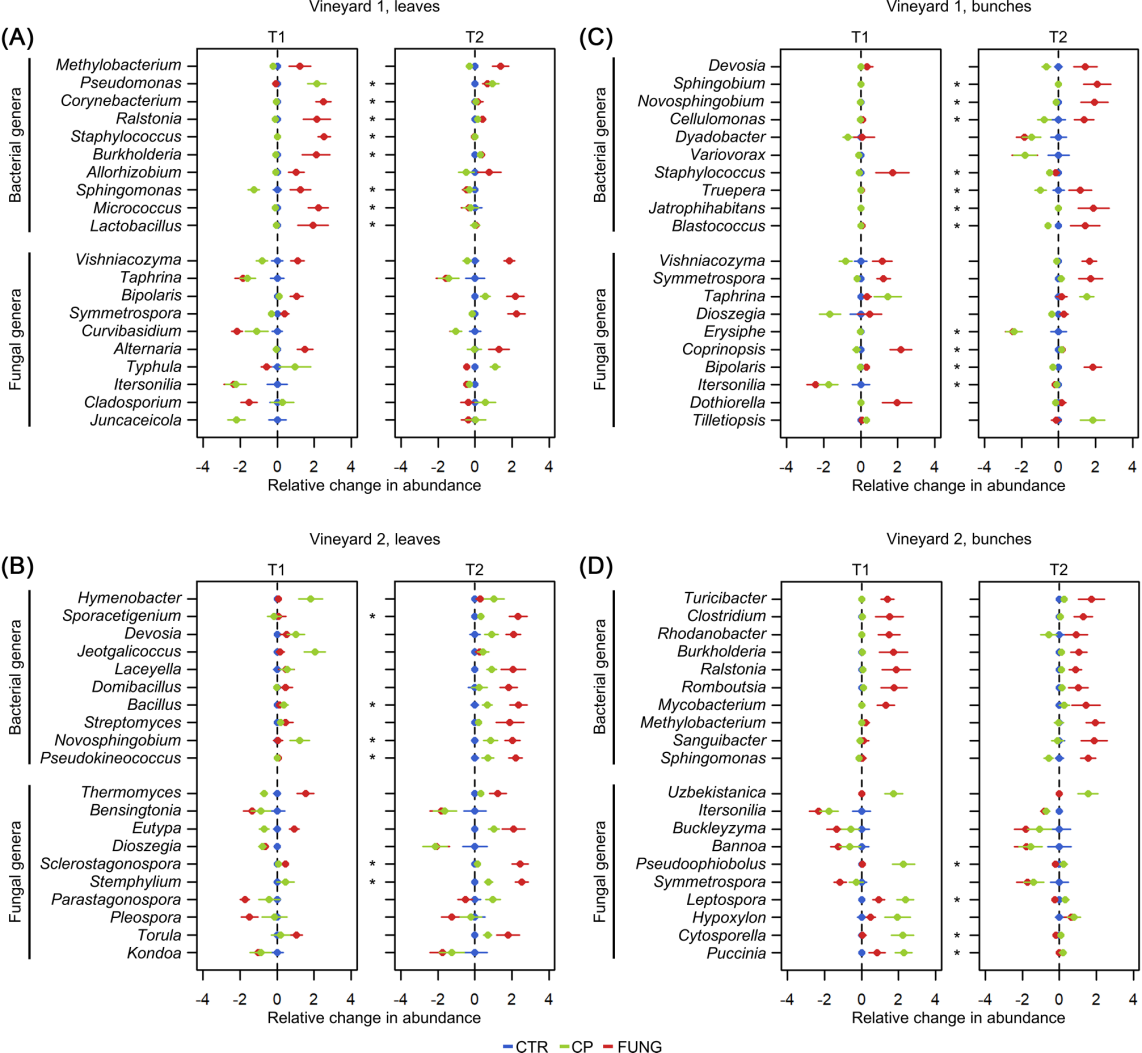
Plant treatments affected the relative abundance of some bacterial and fungal genera of the grapevine phyllosphere. Relative abundances of bacterial and fungal genera were assessed on leaves (A, B) and bunches (C, D) of control plants (CTR, blue) and plants treated with 16 mM choline pelargonate (CP, green) or with a combination of copper and sulfur as reference fungicides (FUNG, red), collected at blooming (T1) and bunch closure (T2) in vineyard 1 and vineyard 2, and relative changes in abundance were calculated as the difference of Z-transformed relative abundances between CP-treated and CTR samples or FUNG-treated and CTR samples. Dots and horizontal bars indicate mean and standard error values of relative changes in abundance, respectively. For each plant compartment and vineyard condition, the ten most responsive bacterial and fungal genera (R^2^ ≥ 0.1; *q ≤ 0.05*) are reported, according to univariate permutational analysis of variance (PERMANOVA with 999; Supplementary Tables S11 and S12). Asterisks indicate significant interaction between treatment and time point (*q ≤ 0.05*). Bacterial and fungal genera are sorted according to decreasing R^2^ values.

On grapevine leaves, the dominant bacterial genera were *Pseudomonas* (10.85%, mean relative abundance), *Hymenobacter* (6.03%), *Acinetobacter* (4.55%), *Sphingomonas* (7.93%), and *Massilia* (5.52%; Fig. 6A, Supplementary Table S11), and the dominant fungal genera were *Aureobasidium* (27.52%), *Sporobolomyces* (19.76%), and *Claviceps* (4.08%; Fig. 6B, Supplementary Table S12). Beta-diversity of bacterial genera (F = 15.88, *P* = 0.001; F= 9.15, *P* = 0.001; and F= 4.05, *P* = 0.001, respectively; Supplementary Table S13A) and fungal genera (F = 96.34, *P* = 0.001; F= 14.37, *P* = 0.001; and F= 5.97, *P* = 0.001, respectively; Supplementary Table S13B) on grapevine leaves was affected by the vineyard, time point, and treatment. In particular, the treatment affected relative abundances of 15 and 13 bacterial genera on grapevine leaves in vineyard 1 and vineyard 2, respectively (Supplementary Table S11), and relative abundances of 24 and 84 fungal genera in vineyard 1 and vineyard 2, respectively (Supplementary Table S12). For each vineyard, 10 bacterial and 10 fungal genera were selected as the most responsive to treatments on grapevine leaves for at least one time point (R^2^ ≥ 0.1; *q ≤ 0.05*; Fig. 7A and B; Supplementary Tables S11 and S12). In vineyard 1, CP treatment increased and decreased the relative abundances of two (*Pseudomonas* and *Typhula*) and six (*Sphingomonas, Vishniacozyma, Taphrina, Curvibasidium, Itersonilia*, and *Juncaceicola*) most responsive genera compared to CTR samples (sorted according to decreasing R^2^ values; Fig. 7A). FUNG treatment increased the relative abundances of *Methylobacterium, Corynebacterium, Ralstonia, Staphylococcus, Burkholderia, Allorhizobium, Sphingomonas, Micrococcus, Lactobacillus, Vishniacozyma, Bipolaris, Symmetrospora*, and *Alternaria*, and decreased the relative abundance of *Taphrina, Curvibasidium, Typhula, Itersonilia*, and *Cladosporium* compared to CTR samples in vineyard 1 (Fig. 7A). In vineyard 2, CP treatment increased the relative abundances of *Hymenobacter, Devosia, Jeotgalicoccus, Laceyella, Bacillus*, and *Novosphingobium*, and decreased the relative abundances of *Bensingtonia, Dioszegia*, and *Kondoa* compared to CTR samples (Fig. 7B). FUNG treatment increased and decreased the relative abundances of thirteen (*Sporacetigenium, Devosia, Laceyella, Domibacillus, Bacillus, Streptomyces, Novosphingobium, Pseudokineococcus, Thermomyces, Eutypa, Sclerostagonospora, Stemphylium*, and *Torula*) and five (*Bensingtonia, Dioszegia, Parastagonospora, Pleospora*, and *Kondoa*) most responsive genera compared to CTR treatment in vineyard 2, respectively (Fig. 7B). Some most responsive genera in vineyard 1 (*Pseudomonas, Corynebacterium, Ralstonia, Staphylococcus, Burkholderia, Sphingomonas, Micrococcus*, and *Lactobacillus*; Fig. 7A) and vineyard 2 (*Sporacetigenium, Bacillus, Novosphingobium, Pseudokineococcus, Sclerostagonospora*, and *Stemphylium*; Fig. 7B) showed a different response to the treatments at T1 and T2.

### Effects of vineyard, time point, and treatment on the taxonomic structure of bacterial and fungal communities of grapevine bunches

Bacterial alpha-diversity of grapevine bunches was mainly affected by the time point, such as Shannon index (PERMANOVA; F = 208.79, *P* = 0.001), number of observed ASVs (F = 261.21, *P* = 0.001), and Pielou’s evenness index (F = 173.04, *P* = 0.001; Supplementary Table S9C). In particular, all bacterial alpha-diversity metrics of grapevine bunches were lower at T1 compared to T2 in both vineyards (Fig. 4C, Supplementary Figs. S6C and S7C). Vineyard was the main driver of fungal alpha-diversity of grapevine bunches, such as Shannon index (F = 79.79, *P* = 0.001), number of observed ASVs (F = 522.49, *P* = 0.001), and Pielou’s evenness index (F = 50.10, *P* = 0.001) (Supplementary Table S9D). Shannon index and number of observed ASVs of fungal communities on grapevine bunches were higher in vineyard 2 compared to vineyard 1 (Fig. 4D, Supplementary Figs. S6D and S7D).

Beta-diversity analysis of bunch samples showed that bacterial communities (Fig. 5C and Supplementary Fig. S8C) and fungal communities (Fig. 5D and Supplementary Fig. S8D) clustered according to vineyard and time point, with a mean reclassification success of 72.0%. Time point and vineyard were the main drivers of beta-diversity for bacterial communities (F = 40.51, *P* = 0.001 and F = 8.06, *P* = 0.001, respectively; Supplementary Table S10C) and fungal communities (F = 42.72, *P* = 0.001 and F = 42.96, *P* = 0.001, respectively; Supplementary Table S10D) on grapevine bunches. Moreover, the treatment affected the beta-diversity of bacterial communities (F = 3.95, *P* = 0.001; Supplementary Table S10C) and fungal communities (F = 4.20, *P* = 0.002; Supplementary Table S10D), and bacterial communities on grapevine bunches differed in FUNG-treated samples compared to CTR and CP-treated samples (Supplementary Table S10C).

The dominant bacterial genera on grapevine bunches were *Pseudomonas* (52.01%, mean relative abundance), *Sphingomonas* (7.62%), and *Massilia* (5.71%; Fig. 6C, Supplementary Table S11), while the dominant fungal genera were *Aureobasidium* (54.64%) and *Sporobolomyces* (10.47%; Fig. 6D, Supplementary Table S12). On grapevine bunches, beta-diversity of bacterial genera (F = 8.11, *P* = 0.001; F= 186.17, *P* = 0.001; and F= 9.18, *P* = 0.001, respectively; Supplementary Table S13C) and fungal genera (F = 12.37, *P* = 0.001; F= 22.19, *P* = 0.001; and F= 6.42, *P* = 0.001, respectively; Supplementary Table S13D) were affected by the vineyard, time point, and treatment. In particular, the treatment affected relative abundances of 34 and 13 bacterial genera on grapevine bunches in vineyard 1 and vineyard 2, respectively (Supplementary Table S11), and relative abundances of 14 and 28 fungal genera in vineyard 1 and vineyard 2, respectively (Supplementary Table S12). For each vineyard, 10 bacterial and 10 fungal genera were selected as the most responsive to treatments on grapevine bunches for at least one time point (R^2^ ≥ 0.1; *q ≤ 0.05*; Fig. 7C and 7D; Supplementary Tables S11 and S12). CP treatment increased the relative abundances of *Taphrina* and *Tilletiopsis*, and decreased the relative abundances of *Cellulomonas, Dyadobacter, Variovorax, Truepera, Blastococcus, Vishniacozyma, Dioszegia, Erysiphe*, and *Itersonilia* compared to CTR samples of grapevine bunches in vineyard 1 (genera sorted according to decreasing R^2^ values; Fig. 7C). FUNG treatment increased and decreased the relative abundances of thirteen (*Devosia, Sphingobium, Novosphingobium, Cellulomonas, Staphylococcus, Truepera, Jatrophihabitans, Blastococcus, Vishniacozyma, Symmetrospora, Coprinopsis, Bipolaris*, and *Dothiorella*) and three (*Variovorax, Erysiphe*, and *Itersonilia*) most responsive genera compared to CTR samples in vineyard 1, respectively (Fig. 7C). In vineyard 2, CP treatment increased the relative abundances of *Uzbekistanica, Pseudophiobolus, Leptospora, Hypoxylon, Cytosporella*, and *Puccinia*, and decreased the relative abundances of *Itersonilia, Buckleyzyma, Bannoa*, and *Symmetrospora* compared to CTR samples (Fig. 7D). FUNG treatment increased and decreased the relative abundance of twelve (*Turcibacter, Clostridium, Rhodanobacter, Burkholderia, Ralstonia, Romboutsia, Mycobacterium, Methylobacterium, Sanguibacter, Sphingomonas, Leptospora*, and *Puccinia*) and four (*Itersonilia, Buckleyzyma, Bannoa*, and *Symmetrospora*) most responsive genera compared to CTR samples in vineyard 2, respectively (Fig. 7D). Some most responsive genera in vineyard 1 (*Sphingobium, Novosphingobium, Cellulomonas, Staphylococcus, Truepera, Jatrophihabitans, Blastococcus, Erysiphe, Coprinopsis, Bipolaris*, and *Itersonilia*; Fig. 7C) and vineyard 2 (*Pseudophiobolus, Leptospora, Cytosporella*, and *Puccinia*; Fig. 7D) showed a different response to the treatments at both T1 and T2.

## Discussion

### Choline pelargonate decreased downy mildew and powdery mildew severity under controlled and field conditions

Downy mildew and powdery mildew are two of the most devastating diseases affecting grapevines, and there is growing interest in developing sustainable alternatives for plant protection (Pirrello et al. 2019, Koledenkova et al. 2022). In this study, we found that CP decreased downy mildew and powdery mildew severity under greenhouse conditions, and the concentration of 16 mM CP resulted in a disease reduction comparable to that achieved with copper- and sulfur-based fungicides used as reference (FUNG), respectively. CP treatment slightly upregulated the expression of some markers of grapevine resistance (Perazzolli et al. 2012) before (*CHIT-3* and *OSM-2*) and one day after (*PR-1*) *P. viticola* inoculation, as a possible partial induction of grapevine defense mechanisms against downy mildew. However, CP showed direct inhibitory effects *in vitro*, against *P. viticola* sporangia, *P. viticola* zoospores, and *E. necator* conidia, and showed no translaminar effects *in planta*, indicating that its mode of action is primarily based on antioomycete and antifungal activities. Likewise, CP has previously demonstrated inhibitory activity against *P. infestans* and *B. cinerea* by altering membrane structure and lipid metabolism *in vitro*, suggesting that its antioomycete and antifungal activities rely mainly on membrane perturbations in phytopathogens (Montanari et al. 2025). Choline carboxylates with carbon-chain lengths greater than eight carbon atoms (e.g. choline pelargonate and choline decanoate) can interact with the bilayer membranes of liposomes (Rengstl et al. 2014), and members of this class of compounds (e.g. choline derivatives of ethanoate, propanoate, butanoate, 2-methylpropanoate, pentanoate, 2,2-dimethylpropanoate, hexanoate, octanoate, and decanoate) can inhibit mycelial growth and spore germination of filamentous fungi, (e.g. *Penicillium brevicompactum, P. corylophilum, P. diversum*, and *P. glandicola*) (Petkovic et al. 2010), corroborating their antifungal properties mediated by membrane perturbations.

Under field conditions, CP decreased downy mildew severity on leaves and bunches compared to control plants, although its efficacy was lower than that of copper- and sulfur-based fungicides, applied as standard practice (FUNG) in organic viticulture in Northern Italy (Dagostin et al. 2010). Weather conditions were less favorable for powdery mildew development, and the efficacy of CP against *E. necator* was comparable to that of copper- and sulfur-based fungicides (FUNG) on leaves and bunches. Other copper alternatives (e.g. biocontrol agents, materials of animal origin, inorganic materials, microbial extracts, and plant extracts) have previously shown limited efficacy against downy mildew under field conditions, possibly due to product sensitivity to environmental factors (e.g. humidity, pH, and UV radiation) and inherent physicochemical characteristics (e.g. scarce adhesion and low rainfastness) (Dagostin et al. 2011, Giovannini et al. 2022), indicating that further studies are required to characterize the efficacy of CP under different environmental conditions and to potentially improve its persistence using appropriate adjuvants. Moreover, the differing efficacy of CP against downy mildew and powdery mildew may be related to the infection biology of the two pathogens. Powdery mildew develops epiphytically on host surfaces (Gadoury et al. 2012), where it is continuously exposed to CP, whereas *P. viticola* develops primarily within the leaf mesophyll (Gessler et al. 2011), where it is likely protected by leaf tissues. In addition, the rainfall sensitivity of CP may further reduce its efficacy against *P. viticola*, especially during periods of intense rainfall that typically coincide with high downy mildew pressure.

### Plant compartment, vineyard, and time point affected the taxonomic structure of bacterial and fungal communities on grapevine leaves and bunches

Dominant bacterial and fungal genera on grapevine leaves and bunches of our study were *Pseudomonas, Hymenobacter, Acinetobacter, Sphingomonas, Massilia, Aureobasidium, Sporobolomyces*, and *Claviceps*, and all of them were previously found as dominant taxa in vineyards (Perazzolli et al. 2014, Singh et al. 2019, Swift et al. 2021, Bettenfeld et al. 2022, Swift et al. 2023, Di Gianvito et al. 2025). In particular, some dominant bacteria and fungi may be associated with beneficial functions on grapevine plants, such as plant growth promotion (e.g. *Methylobacterium*), or biocontrol activity (e.g. *Sphingomonas* and *Aureobasidium*) (Singh et al. 2019).

As previously reported (Castañeda et al. 2018, Vitulo et al. 2019, Swift et al. 2021, Bettenfeld et al. 2022, Swift et al. 2023, Steng et al. 2024, Legesse et al. 2025), we found major effects of the plant compartment on the composition of bacterial and fungal communities, and data from leaves and bunches were analyzed separately. Moreover, alpha-diversity and beta-diversity analyses (based on both ASVs and genus-level aggregated ASVs) revealed that the taxonomic structure of bacterial and fungal communities on grapevine leaves and bunches was mainly influenced by vineyard and time point, indicating that environmental conditions and phenological stages shape the grapevine phyllosphere microbiota. Similarly, vineyard and sampling time have been reported to affect the composition of bacterial and fungal communities associated with grapevine leaves in California (Yang et al. 2024) and northern Italy (Perazzolli et al. 2014, Perazzolli et al. 2020). In addition, sampling time has been shown to influence the taxonomic structure of fungal communities on grapevine leaves in South Africa (Gobbi et al. 2020), as well as bacterial and fungal communities on grapevine bunches in Germany (Kecskeméti et al. 2016) and in Portugal (Stahl et al. 2022), supporting the strong contribution of temporal and environmental factors on phyllosphere micro-organisms.

### Choline pelargonate and reference fungicides affected the relative abundance of specific bacterial and fungal taxa on grapevine leaves and bunches

Treatments with CP and reference fungicides (copper and sulfur; FUNG) showed only slight effects on alpha-diversity and beta-diversity (based on both ASVs and genus-level aggregated ASVs) of leaf- and bunch-associated microbial communities. In particular, bacterial communities on grapevine leaves and bunches differed in FUNG-treated samples compared to CTR and CP-treated samples, indicating a general effect of FUNG and a negligible effect of CP on microbial assemblages. Similarly, impacts on bacterial communities associated with grapevine leaves have been reported to be less evident for biological fungicides (*B. subtilis* OCASB12) than chemical fungicides (pyraclostrobin and myclobutanil) (He et al. 2024). Biological and chemical fungicides previously showed only slight effects on the diversity of bacterial and fungal communities associated with grapevine leaves and bunches (Perazzolli et al. 2014, Kecskeméti et al. 2016, Gobbi et al. 2020, Perazzolli et al. 2020, Yang et al. 2024), corroborating the resilience of the phyllosphere microbiota, possibly due to microbial colonization of ecological niches, which are less influenced by fungicide treatments.

Although no global effects on bacterial and fungal communities were found, CP and FUNG treatments can affect the relative abundances of some specific taxa of the grapevine phyllosphere, in agreement with previous findings with biological and chemical fungicides (Perazzolli et al. 2014, Kecskeméti et al. 2016, Castañeda et al. 2018, Gobbi et al. 2020, Perazzolli et al. 2020, Rantsiou et al. 2020, He et al. 2024, Steng et al. 2024). In particular, CP treatment affected the relative abundances of 22 bacterial genera and 52 fungal genera, while FUNG treatment affected the relative abundances of 36 bacterial genera and 83 fungal genera in leaves and bunches across both vineyards and time points, indicating broader shifts caused by FUNG than CP treatments. In particular, the relative abundances of specific genera were affected by both CP and FUNG treatments on leaves of vineyard 1 (decreases of *Curvibasidium, Erysiphe, Incrucipulum, Itersonilia, Lophium*, and *Taphrina*), leaves of vineyard 2 (increases of *Bacillus, Devosia, Laceyella*, Bipolaris, and Entyloma; decreases of: *Bensingtonia, Buckleyzyma, Dioszegia, Kondoa*, and *Symmetrospora*), bunches of vineyard 1 (decreases of *Erysiphe*), and bunches of vineyard 2 (increases of *Botryosphaeria, Coprinopsis*, and *Hypoxylon*; decreases of *Bannoa, Buckleyzyma, Farysia, Itersonilia*, and *Symmetrospora*). Moreover, CP treatment (9 and 12 genera on leaves and bunches, respectively) and FUNG treatment (11 and 6 genera on leaves and bunches, respectively) decreased the relative abundances of most responsive bacterial and fungal genera compared to CTR samples across both vineyards and time points. In agreement with the decrease of powdery mildew severity on CP-treated and FUNG-treated leaves and bunches, the relative abundance of *Erysiphe* genus decreased in vineyard 1. Moreover, CP and FUNG treatments decreased the relative abundances of genera comprising potential crop pathogens, such as *Taphrina* spp. (Christita et al. 2022), *Itersonilia* spp. (Chappell et al. 2024), and *Cladosporium* spp. (El-Dawy et al. 2021). Conversely, the relative abundances of other most responsive bacterial and fungal genera increased on CP-treated (nine and eight genera, respectively) and FUNG-treated (26 and 23 genera, respectively) leaves and bunches compared to CTR samples across both vineyards and time points. In particular, CP and FUNG treatments increased the relative abundances of bacterial and fungal genera comprising potential biocontrol agents against crop pathogens [e.g. *Tilletiopsis* spp. (Richter et al. 2019), *Hypoxylon* spp. (Jin et al. 2022), *Alternaria* spp.(González and Tello 2011), *Bacillus* spp. (Legein et al. 2020), and *Burkholderia* spp. (Elshafie and Camele 2021)] and potential beneficial micro-organisms that can influence plant health and production [e.g. *Pseudomonas* spp. (Zarraonaindia et al. 2015), *Methylobacterium* spp. (Zarraonaindia et al. 2015), *Devosia* spp. (Wicaksono et al. 2023), *Sphingobium* spp. and *Novosphingobium* spp. (Boss et al. 2022)], suggesting a partial engineering of the phyllosphere microbiota with changes in the proportion between beneficial and pathogenic plant-associated micro-organisms. However, DNA of dead micro-organisms can be detected by amplicon sequencing analysis (Carini et al. 2016), indicating that further functional studies are required to clarify the possible selectivity of CP in the perturbation of bacterial, fungal, or oomycete membranes, as well as its differential impact on phytopathogenic and beneficial micro-organisms.

In this study, we used rarefaction followed by univariate PERMANOVA to prioritize robust control over sequencing depth artifacts and data sparsity (Schloss Patrick 2024). However, the analysis of relative abundance data without compositional transformations (such as log-ratio frameworks used in model-based approaches like ANCOM-BC2) (Lin and Peddada 2024) are affected by the inherent constraint of mathematical closure, meaning that shifts in the relative abundances of one taxon can influence the relative abundances of other taxa (Gloor et al. 2017). While the permutation-based approach used in our analysis avoids rigid distributional assumptions, linear model-based frameworks could offer advantages in directly estimating absolute bias corrections and log-fold changes (Lin and Peddada 2024). Thus, future multi-omics or absolute quantification studies (e.g. using spike-in controls) combined with model-based approaches will be valuable for validating taxonomic shifts without the constraints imposed by the compositional nature of the data. Moreover, amplicon sequencing analysis allowed taxonomic annotation only at the genus level (Gupta et al. 2019), and several genera can include both phytopathogenic and beneficial species. Thus, potential ecological roles of plant-associated micro-organisms cannot be easily assigned, and further shotgun metagenomic, long-read sequencing, and microbial cultivation coupled to genomic analyses are required to better identify microbial taxa and gene functions affected by CP treatments on different grapevine cultivars and environmental conditions.

## Conclusions

CP effectively decreased the severity of downy mildew and powdery mildew on grapevine plants under greenhouse and field conditions. CP showed direct inhibitory effects on *P. viticola* and *E. necator*, with slight induction of grapevine defense mechanisms and no translaminar effects, indicating that its mode of action is mainly based on direct antioomycete and antifungal activities. Plant compartment, vineyard location, and sampling time were the main drivers of microbial community structure. CP treatment partially modified the relative abundances of specific bacterial and fungal taxa without affecting the overall microbial diversity on grapevine leaves and bunches. Reference fungicides of organic viticulture (copper and sulfur) caused broader shifts compared to CP treatment in terms of the number of responsive genera. Thus, CP had negligible side effects on the grapevine phyllosphere microbiota, highlighting its potential as a sustainable disease management approach that preserves indigenous bacterial and fungal communities of leaves and bunches. However, further shotgun metagenomic, long-read sequencing, and microbial cultivation analyses are required to better assess the effects of CP treatments on beneficial and phytopathogenic micro-organisms analyzing different grapevine cultivars environmental conditions. Moreover, further studies on CP formulations and persistence under field conditions are required to develop complementary strategies to conventional fungicides.

## Supplementary Material

fiag082_Supplemental_Files

## Data Availability

Amplicon sequencing reads were deposited at the Sequence Read Archive of the NCBI (https://www.ncbi.nlm.nih.gov/sra) under the BioProject number PRJNA1200613.
