# Supplementary material for: Choline pelargonate treatments decreased downy mildew and powdery mildew symptoms with negligible effects on grapevine phyllosphere micro-organisms
Source: FEMS Microbiol Ecol. 2026 Jul 24;102(8):fiag082. doi: 10.1093/femsec/fiag082 (PMC13436970; doi:10.1093/femsec/fiag082)
Supplement: Supplementary file 1 [file fiag082_supplemental_files.zip › Montanari et al Supplementary Figures.pdf]

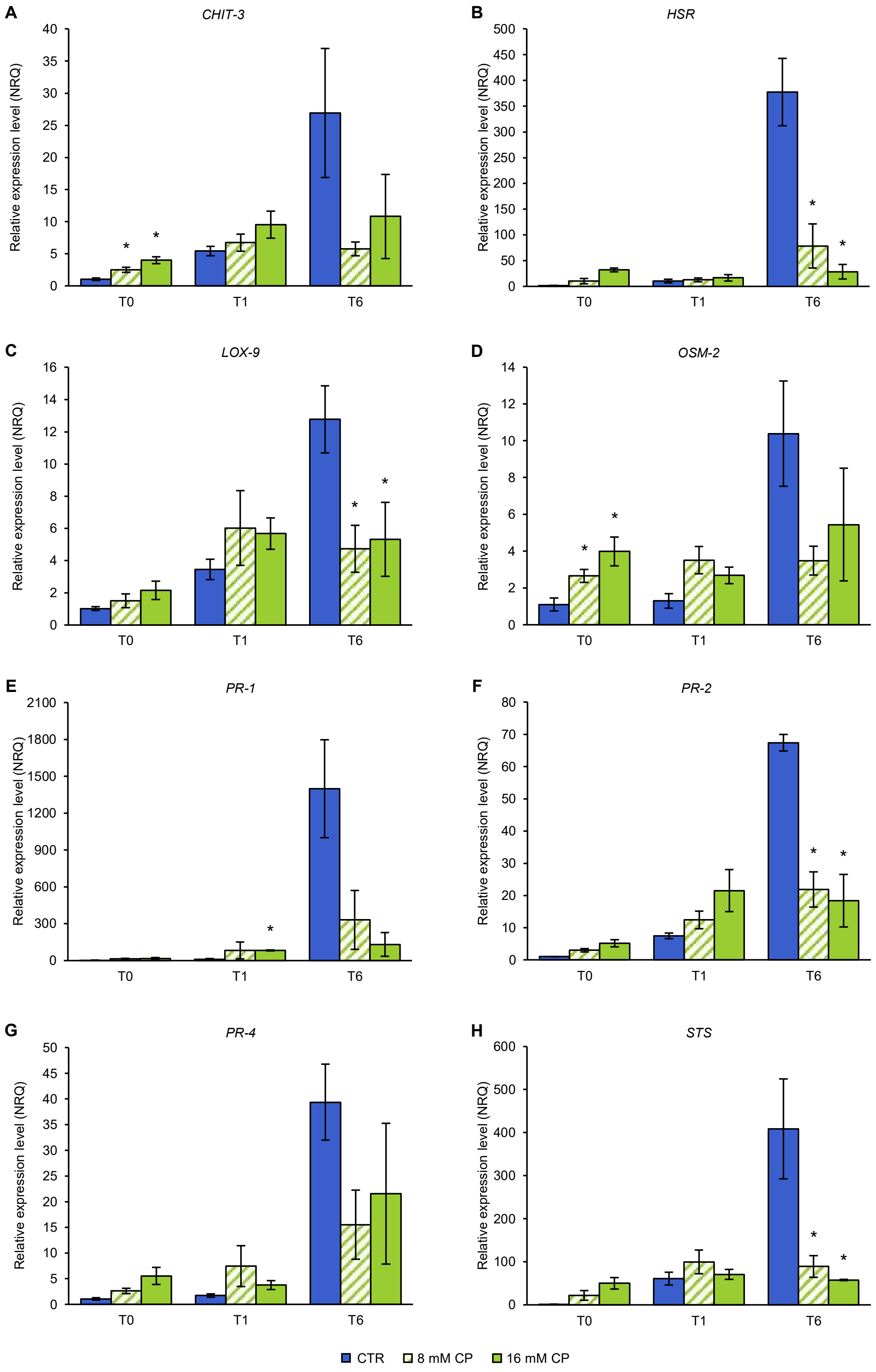

Supplementary Figure S1. Choline pelargonate caused slight changes in the expression of defense-related genes under greenhouse conditions. Plants were treated with water (blue; control, CTR), 8 mM (stripped bars), or 16 mM (solid bars) choline pelargonate (CP; green) to both surfaces of each leaf (whole leaf treatment), and leaf samples were collected just before inoculation (T0) or at one (T1) and six (T6) days post inoculation with *Plasmopara viticola*. The expression of genes encoding chitinase 3 (*CHIT-3*, A), hypersensitive response-related gene (*HSR*, B), lipoxygenase 9 (*LOX-9*, C),

osmotin 2 (*OSM-2*, D), pathogenesis-related (PR) protein 1 (*PR-1*, E), protein 2 (*PR-2*, F), and protein 4 (*PR-4*, G), and stilbene synthase (*STS*, H) was expressed as normalized relative quantity (NRQ) to CTR plants at T0 using *actin* and *VATP16* as housekeeping genes for data normalization. Two independent experiments were carried out, and similar expression profiles were observed. Mean and standard error values of three biological replicates (plants) of one representative experiment are reported for each treatment and time point. For each time point, asterisks indicate significant differences between CP-treated and CTR plants, according to the Student's t-test ($P \leq 0.05$).

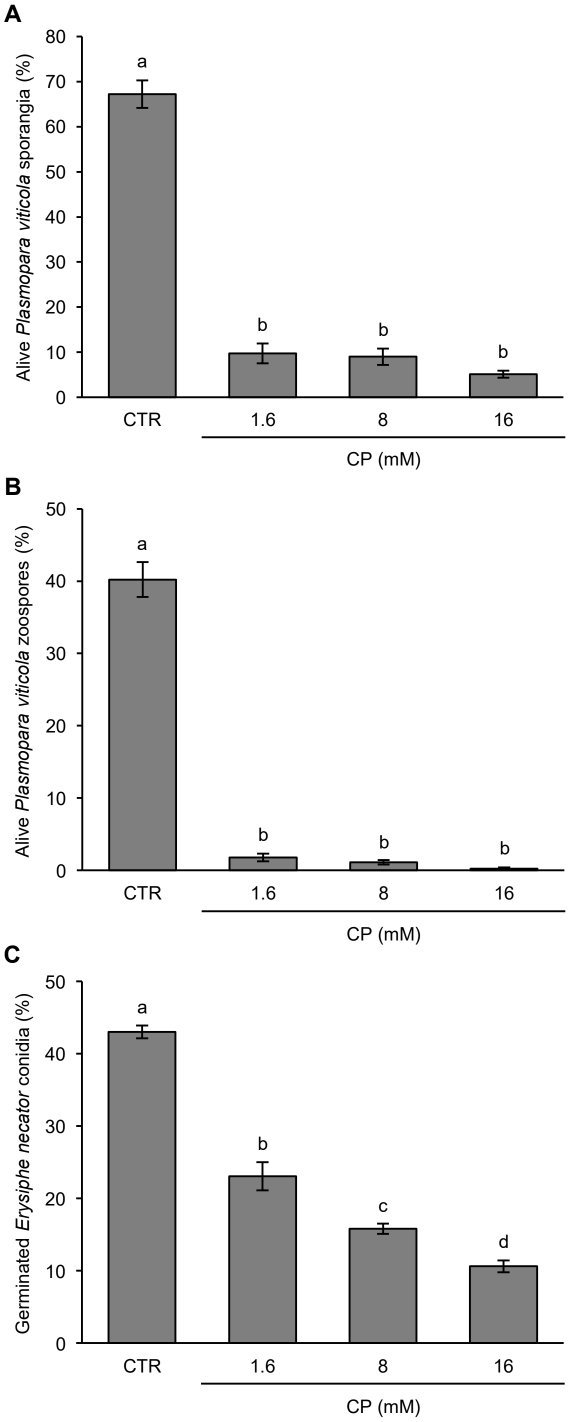

Supplementary Figure S2. Choline pelargonate decreased *Plasmopara viticola* and *Erysiphe necator* viability. A suspension of *P. viticola* sporangia (1×10^6 sporangia/mL) was supplemented or not (control; CTR) with 1.6 mM, 8 mM, or 16 mM choline pelargonate (CP), and the percentage (%) of alive sporangia (A; empty sporangia) and alive zoospores (B; motile zoospores) was assessed after 1 h and 5 min of incubation, respectively. Grapevine leaf disks were treated with water (control; CTR), 1.6 mM, 8 mM, or 16 mM CP, and the percentage (%) of germinated conidia was assessed 48 h after *E. necator* inoculation (C). Comparable results were obtained between the two experiments (Kruskal-Wallis $P > 0.05$), and data were pooled. Mean and standard error values of twelve biological replicates (tubes; A and B) or 24 biological replicates (leaf disks; C) from the experiments are reported for each treatment. Different letters indicate significant differences among treatments, according to the Kruskal-Wallis test followed by the Dunn's test ($P \leq 0.05$).

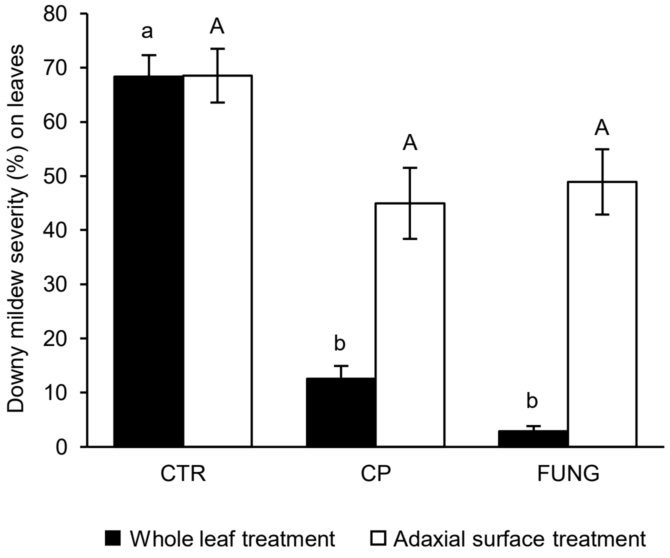

Supplementary Figure S3. Choline pelargonate showed no translaminar effect against downy mildew under greenhouse conditions. Plants were treated with water (control; CTR), 16 mM choline pelargonate (CP), or copper as reference fungicide (FUNG) on both surfaces of each leaf (whole leaf treatment, black) or only on the adaxial surface of each leaf (adaxial surface treatment, white). Downy mildew severity was assessed as the percentage of leaf area with disease symptoms at seven days post inoculation. Comparable results were obtained between the two experiments (Kruskal-Wallis $P > 0.05$), and data were pooled. Mean and standard error values of twelve biological replicates (plants) from two experiments are reported for each treatment. Different lowercase and uppercase letters indicate significant differences among treatments for whole leaf treatment and adaxial surface treatment, according to the Kruskal-Wallis test followed by the Dunn's test ($P \leq 0.05$), respectively.

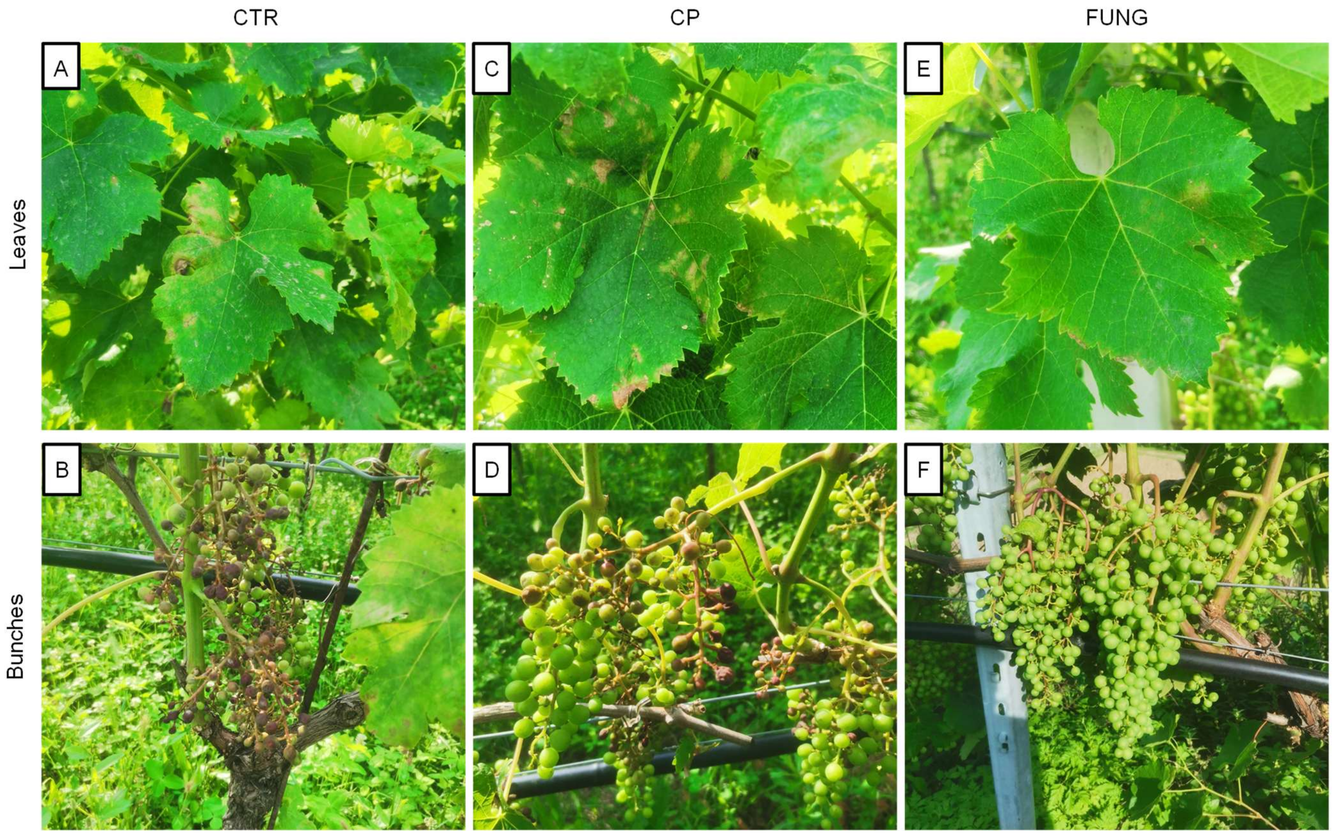

Supplementary Figure S4. Downy mildew severity under field conditions. Downy mildew severity was assessed on leaves and bunches of control plants (CTR; A, B) and plants treated with 16 mM choline pelargonate (CP; C, D) or with a combination of copper and sulfur as reference fungicides (FUNG; E, F) in vineyard 1. Four plots of twelve plants each were analyzed for each treatment in a randomized block design, and downy mildew severity was assessed as the percentage (%) of leaf area or bunch area with downy mildew symptoms on 60 leaves or 60 bunches per plot. Pictures were taken at bunch closure (1st July 2024, BBCH-77)

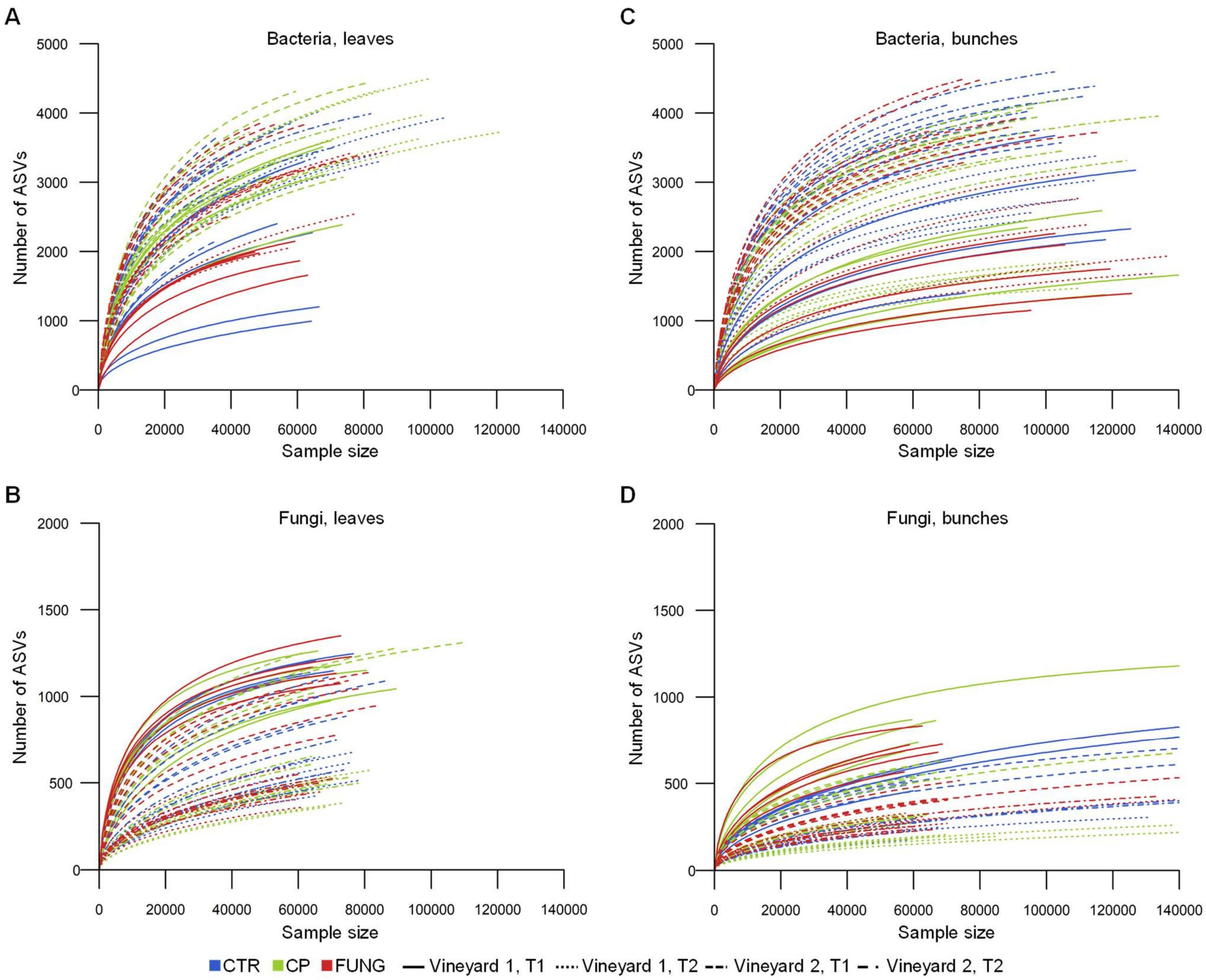

Supplementary Figure S5. Rarefaction curves of sequencing data. Rarefaction curves of bacterial and fungal communities on grapevine leaves (A, B) and bunches (C, D) of control plants (CTR; blue) and plants treated with 16 mM choline pelargonate (CP; green) or with a combination of copper and sulfur as reference fungicides (FUNG; red) are reported for samples collected from two vineyards (vineyard 1 and vineyard 2) at blooming (T1; solid line vineyard 1 and dashed line vineyard 2) and bunch closure (T2; dotted line vineyard 1 and dot-dashed line vineyard 2) in five biological replicates.

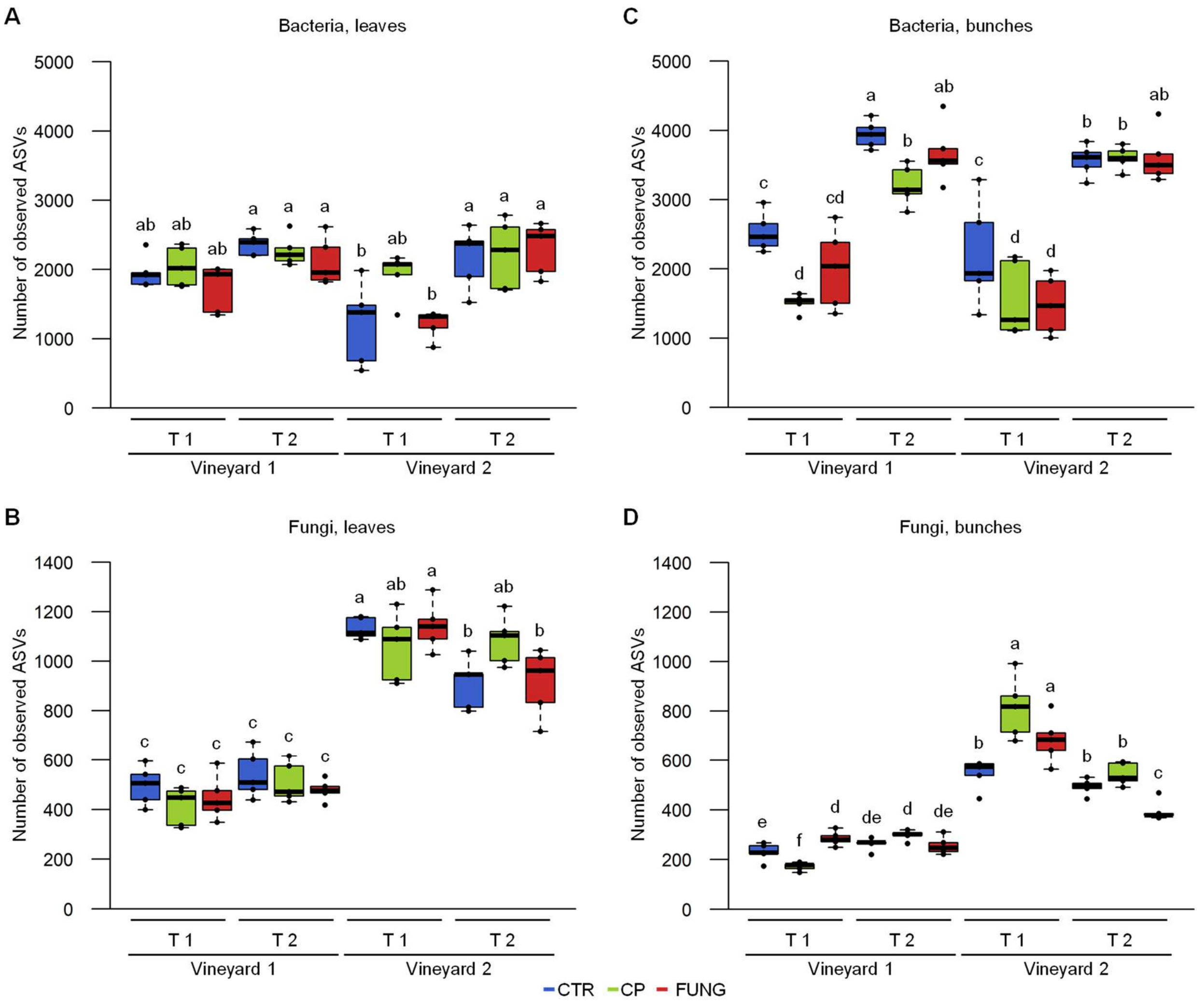

Supplementary Figure S6. The number of observed amplicon sequence variants (ASVs) of grapevine phyllosphere microorganisms was affected by vineyard, time point, and/or treatment. The number of bacterial and fungal ASVs was assessed on leaves (A, B) and bunches (C, D) of control plants (CTR; blue) and plants treated with 16 mM choline pelargonate (CP; green) or with a combination of copper and sulfur as reference fungicides (FUNG; red), collected at blooming (T1) and bunch closure (T2) in vineyard 1 and vineyard 2. The effects of vineyard, time point, and treatment were evaluated using univariate permutational analysis of variance (PERMANOVA with 999 permutations; Supplementary Table S8). Different letters indicate significant differences among samples, according to pairwise PERMANOVA (999 permutations; $P \leq 0.05$).

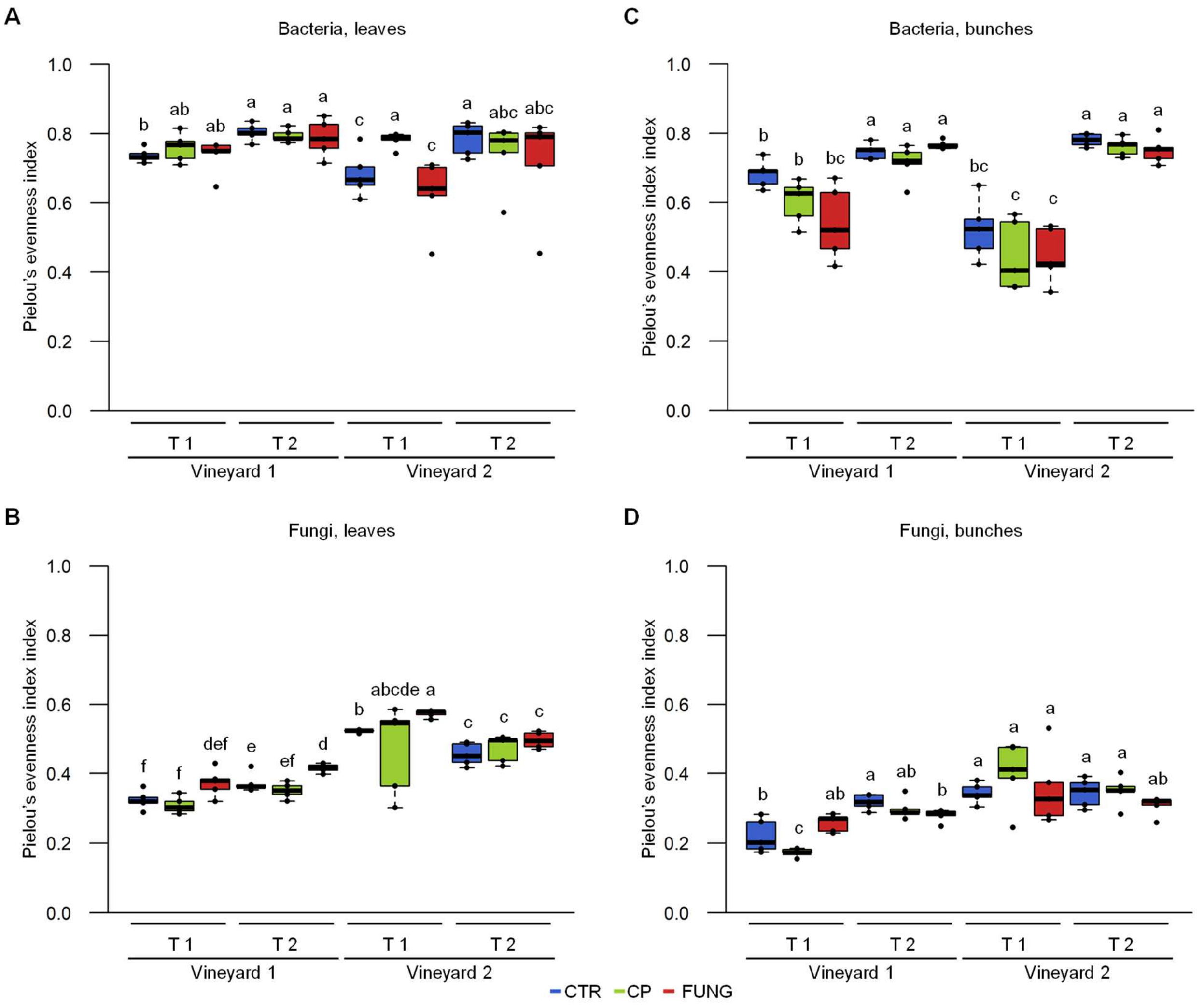

Supplementary Figure S7. The evenness index of grapevine phyllosphere microorganisms was affected by vineyard, time point, and/or treatment. Pielou's evenness index of bacterial and fungal communities was assessed on leaves (A, B) and bunches (C, D) of control plants (CTR; blue) and plants treated with 16 mM choline pelargonate (CP; green) or with a combination of copper and sulfur as reference fungicides (FUNG; red), collected at blooming (T1) and bunch closure (T2) in vineyard 1 and vineyard 2. The effects of vineyard, time point, and treatment were evaluated using univariate permutational analysis of variance (PERMANOVA; Supplementary Table S8). Different letters indicate significant differences among samples, according to pairwise PERMANOVA (999 permutations; $P \leq 0.05$).

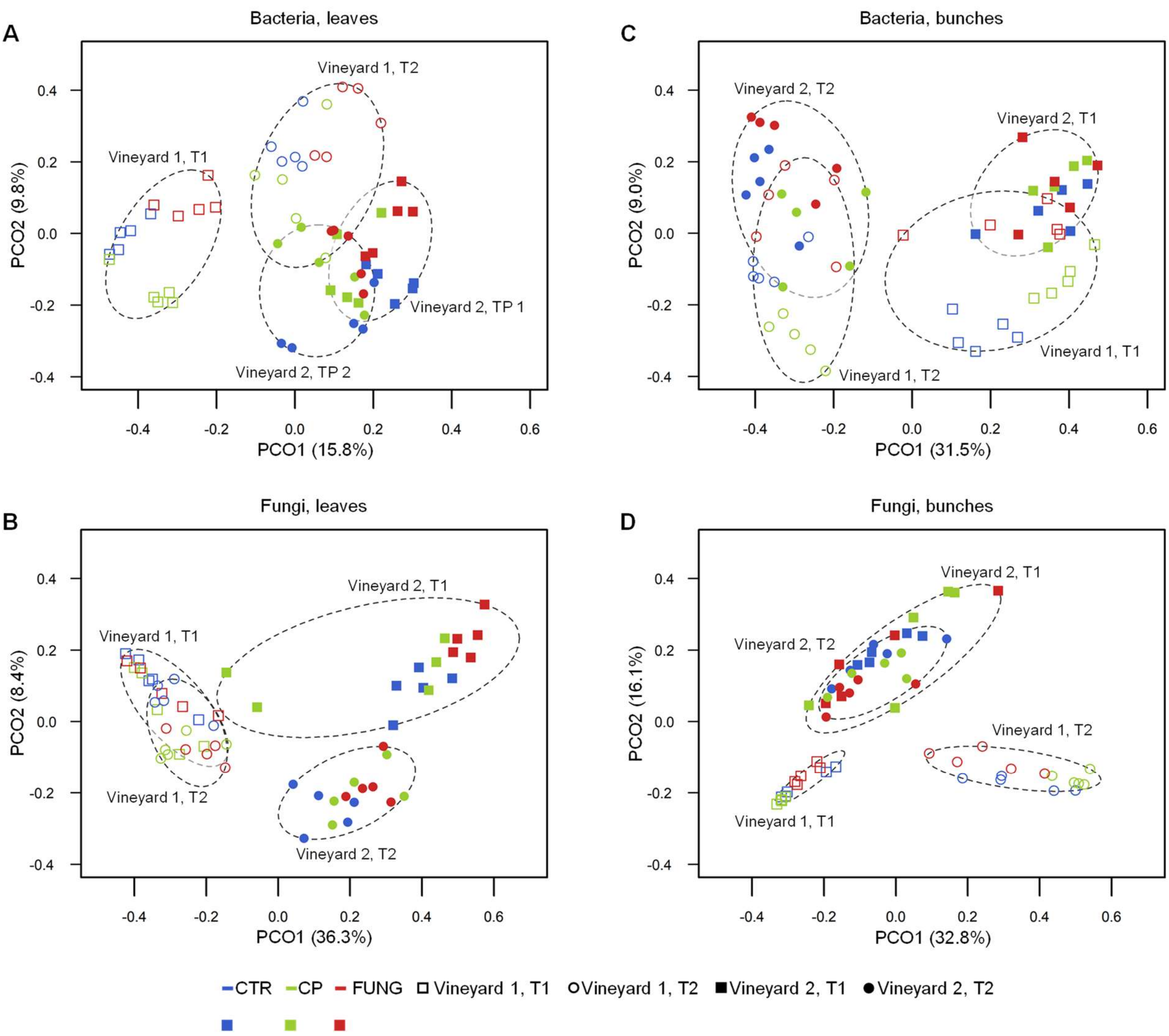

Supplementary Figure S8. Principal coordinate analysis (PCoA) of grapevine phyllosphere microorganisms. PCoA was obtained for bacterial and fungal communities of leaves (A, B) and bunches (C, D) collected from control plants (CTR; blue) and plants treated with 16 mM choline pelargonate (CP; green) or with a combination of copper and sulfur as reference fungicides (FUNG; red), at blooming (T1; square symbols) and bunch closure (T2; circle symbols), in vineyard 1 (empty symbols) and vineyard 2 (solid symbols) using the Bray-Curtis dissimilarity matrices. The effects of vineyard, time point, and treatment were evaluated using multivariate permutational analysis of variance (PERMANOVA with 999 permutations; Supplementary Table S9). Dashed ellipses enclose samples from the same vineyard and time point, and percentages indicate the reclassification success of samples.

Supplementary Table S1. Choline pelargonate decreased downy mildew severity on grapevine leaf discs.
Leaf discs were treated with distilled water (control; CTR), or with 0.2 mM, 0.3 mM, 0.8 mM, 1.6 mM, 8 mM, or 16 mM choline pelargonate (CP). Disease reduction (efficacy) was calculated according to the following equation: (disease severity of CTR leaf disc – disease severity of CP-treated leaf disc) / disease severity of CTR disc × 100. Mean and standard error values of five biological replicates (dishes with five disks each) are reported for each treatment. Different letters indicate significant differences among treatments, according to the Kruskal-Wallis test followed by the Dunn's test ($P \leq 0.05$).

Supplementary Table S2. Primer sequences of grapevine genes analyzed by quantitative real-time PCR.
Gene name (column A), gene abbreviation (column B), and accession numbers of the National Center for Biotechnology Information (NCBI) database (http://www.ncbi.nlm.nih.gov; column C) are reported for the selected grapevine genes. Sequences of the forward primer (column D) and reverse primer (column E) are indicated for each gene with the corresponding bibliographic reference (column F).

Supplementary Table S3. Disease management data of the 2024 season.
The time points of sampling and disease assessments are reported in columns B-D and N-P for vineyard 1 and vineyard 2, respectively. Disease management, including the treatment date, phenological stage, aim of treatment, product name, concentration (mL/L), and applied quantity (L/ha), is reported in columns E-M and Q-Y for vineyard 1 and vineyard 2, respectively.

Supplementary Table S4. Amplification conditions for bacterial 16S and fungal ITS fragments.
The bacterial V5-V7 region of 16S ribosomal rRNA gene and the fungal ITS2 region of the internal transcribed spacer (ITS) fragment were amplified from 5 ng template DNA with a nested PCR approach. Reagents (FastStart High-Fidelity PCR system), amplification cycles, and primers used in the first and second amplification are reported for bacterial 16S and fungal ITS.

Supplementary Table S5. The plant compartment influences the beta-diversity of grapevine phyllosphere microorganisms.
Beta-diversity of bacterial (A) and fungal (B) communities was assessed on leaves and bunches of control plants (CTR) and plants treated with 16 mM choline pelargonate (CP) or with a combination of copper and sulfur as reference fungicides (FUNG), collected at blooming (T1) and bunch closure (T2) in vineyard 1 and vineyard 2. The effects of plant compartment, vineyard, time point, treatment, and their interactions were evaluated using multivariate permutational analysis of variance (PERMANOVA) with 999 permutations, and explained variance (R^2), F-ratio (F), and *p*-value (*p*) results are reported (lines 7-15). The homogeneity of group dispersions was tested with permutational analysis of multivariate dispersions (PERMDISP) with 999 permutations (lines 19-43). The levels of significance are indicated by asterisks ($P \leq 0.05$: *; $P \leq 0.01$: **; $P \leq 0.001$: ***).

Supplementary Table S6. Sequencing results of bacterial (A) and fungal (B) communities on grapevine leaves and bunches.
Numbers of raw reads, filtered reads, observed amplicon sequence variants (ASVs), Pielou's evenness index, and Shannon index are reported for bacterial (16S; A) and fungal (ITS; B) communities on grapevine leaves (Leaf) and bunches (Bunch) of control plants (CTR) and plants treated with 16 mM choline pelargonate (CP) or with a combination of copper and sulfur as

reference fungicides (FUNG) collected in five biological replicates (named a, b, c, d, e) at blooming (T1) and bunch closure (T2) in vineyard 1 (Vin1) and vineyard 2 (Vin2).

Supplementary Table S7. Bacterial amplicon sequence variants (ASVs) of the grapevine phyllosphere.
Bacterial ASVs (column A), were assessed on leaves (Leaf) and bunches (Bunch) of control plants (CTR) and plants treated with 16 mM choline pelargonate (CP) or with a combination of copper and sulfur as reference fungicides (FUNG) collected in five biological replicates (named a, b, c, d, e) at blooming (T1) and bunch closure (T2) in vineyard 1 (Vin1) and vineyard 2 (Vin2). For each ASV (column A), taxonomic annotation (columns B-H), mean and standard error values of relative abundances in grapevine leaf samples (columns I-AF) and bunch samples (columns EW-FT) are reported together with rarefied sequence read counts (columns AG-CN and FU-IB, respectively) and relative abundances (columns CO-EV and IC-KJ, respectively) of each replicate.

Supplementary Table S8. Fungal amplicon sequence variants (ASVs) of the grapevine phyllosphere.
Fungal ASVs (column A) were assessed on leaves (Leaf) and bunches (Bunch) of control plants (CTR) and plants treated with 16 mM choline pelargonate (CP) or with a combination of copper and sulfur as reference fungicides (FUNG) collected in five biological replicates (named a, b, c, d, e) at blooming (T1) and bunch closure (T2) in vineyard 1 (Vin1) and vineyard 2 (Vin2). For each ASV (column A), taxonomic annotation (columns B-H), mean and standard error values of relative abundances in grapevine leaf samples (columns I-AF) and bunch samples (columns EW-FT) are reported together with rarefied sequence read counts (columns AG-CN and FU-IB, respectively) and relative abundances (columns CO-EV and IC-KJ, respectively) of each replicate.

Supplementary Table S9. Factors affecting alpha-diversity metrics of grapevine phyllosphere microorganisms.
Alpha-diversity metrics [Shannon index, number of amplicon sequence variants (ASVs), and Pielou's evenness index] of bacterial (A, C) and fungal (B, D) communities was assessed on leaves (A, B) and bunches (C, D) of control plants (CTR) and plants treated with 16 mM choline pelargonate (CP) or with a combination of copper and sulfur as reference fungicides (FUNG), collected at blooming (T1) and bunch closure (T2) in vineyard 1 and vineyard 2. For each alpha-diversity metric, the effects of vineyard, time point, treatment, and their interactions were evaluated using univariate permutational analysis of variance (PERMANOVA) with 999 permutations, and explained variance (R^2), F-ratio (F), and *p*-value (*p*) results are reported (lines 7-43). The homogeneity of group dispersions was tested with permutational analysis of multivariate dispersions (PERMDISP) with 999 permutations (lines 47-98). The levels of significance are indicated by asterisks ($P \leq 0.05$: *; $P \leq 0.01$: **; $P \leq 0.001$: ***).

Supplementary Table S10. Factors affecting the beta-diversity of grapevine phyllosphere microorganisms.
Beta-diversity metrics of bacterial (A, C) and fungal (B, D) communities was assessed on leaves (A, B) and bunches (C, D) of control plants (CTR) and plants treated with 16 mM choline pelargonate (CP) or with a combination of copper and sulfur as reference fungicides (FUNG), collected at blooming (T1) and bunch closure (T2) in vineyard 1 and vineyard 2. The effects of vineyard, time point, treatment, and their interactions were evaluated using multivariate permutational analysis of variance (PERMANOVA) with 999 permutations, and explained variance (R^2), F-ratio (F), and *p*-value (*p*) results are reported (lines 7-18). The homogeneity of group dispersions was tested with permutational analysis of multivariate dispersions (PERMDISP) with 999 permutations (lines 22-37). Pairwise comparisons between CTR, CP-treated, and FUNG-treated samples were carried out

with pairwise PERMANOVA with 999 permutations, and explained variance (R^2), F-ratio (F), and *p*-value (*p*) results are reported (lines 41-46). The levels of significance are indicated by asterisks (*P ≤ 0.05*: *; *P ≤ 0.01*: **; *P ≤ 0.001*: ***).

Supplementary Table S11. Effects of treatments on the relative abundances of bacterial genera of the grapevine phyllosphere.
Read counts of each ASV assigned to the same bacterial genus (column A) were aggregated, and relative abundances were assessed on leaves and bunches of control plants (CTR) and plants treated with 16 mM choline pelargonate (CP) or with a combination of copper and sulfur as reference fungicides (FUNG), collected at blooming (T1) and bunch closure (T2) in vineyard 1 and vineyard 2. For each genus, the mean relative abundance, mean Z-transformed relative abundance, and the corresponding standard error values are reported for each plant compartment, vineyard, and time point (columns B-Y, AM-BJ, BX-CU, and DI-EF).
For each genus, explained variance (R^2), F-ratio (F), *p*-value (*p*), and *q*-value (*q*) results of univariate permutational analysis of variance (PERMANOVA) with 999 permutations are reported to identify genera whose relative abundance was affected by treatments (responsive genera; *q*-value ≤ 0.05). The interaction between treatment and time point was also evaluated to highlight genera with different responses to the treatments at T1 and T2 (*q*-value ≤ 0.05, columns Z-AF and BK-BQ for leaf samples of vineyard 1 and vineyard 2, respectively; columns CV-DB and EG-EM for bunch samples of vineyard 1 and vineyard 2, respectively).
For each responsive genus, pairwise PERMANOVA with 999 permutations was used to identify significant changes in relative abundances between CTR, CP-treated, and FUNG-treated samples (columns AG-AL and BR-BW for leaf samples of vineyard 1 and vineyard 2, respectively; columns DC-DH and EN-ES for bunch samples of vineyard 1 and vineyard 2, respectively).
The level of significance is indicated by asterisks (*P ≤ 0.05*: *; *P ≤ 0.01*: **; *P ≤ 0.001*: ***).

Supplementary Table S12. Effects of treatments on the relative abundances of fungal genera of the grapevine phyllosphere.
Read counts of each ASV assigned to the same fungal genus (column A) were aggregated, and relative abundances were assessed on leaves and bunches of control plants (CTR) and plants treated with 16 mM choline pelargonate (CP) or with a combination of copper and sulfur as reference fungicides (FUNG), collected at blooming (T1) and bunch closure (T2) in vineyard 1 and vineyard 2. For each genus, the mean relative abundance, mean Z-transformed relative abundance, and the corresponding standard error values are reported for each plant compartment, vineyard, and time point (columns B-Y, AM-BJ, BX-CU, and DI-EF).
For each genus, explained variance (R^2), F-ratio (F), *p*-value (*p*), and *q*-value (*q*) results of univariate permutational analysis of variance (PERMANOVA) with 999 permutations are reported to identify genera whose relative abundance was affected by treatments (responsive genera; *q*-value ≤ 0.05). The interaction between treatment and time point was also evaluated to highlight genera with different responses to the treatments at T1 and T2 (*q*-value ≤ 0.05, columns Z-AF and BK-BQ for leaf samples of vineyard 1 and vineyard 2, respectively; columns CV-DB and EG-EM for bunch samples of vineyard 1 and vineyard 2, respectively).
For each responsive genus, pairwise PERMANOVA with 999 permutations was used to identify significant changes in relative abundances between CTR, CP-treated, and FUNG-treated samples (columns AG-AL and BR-BW for leaf samples of vineyard 1 and vineyard 2, respectively; columns DC-DH and EN-ES for bunch samples of vineyard 1 and vineyard 2, respectively).
The level of significance is indicated by asterisks (*P ≤ 0.05*: *; *P ≤ 0.01*: **; *P ≤ 0.001*: ***).

Supplementary Table S13. Factors affecting the beta-diversity based on the relative abundances of bacterial and fungal genera.

Read counts of each ASV assigned to the same genus were aggregated, and relative abundances of bacterial and fungal genera were assessed on leaves (A, B) and bunches (C, D) of control plants (CTR) and plants treated with 16 mM choline pelargonate (CP) or with a combination of copper and sulfur as reference fungicides (FUNG), collected at blooming (T1) and bunch closure (T2) in vineyard 1 and vineyard 2. The effects of vineyard, time point, treatment, and their interactions were evaluated using multivariate permutational analysis of variance (PERMANOVA) with 999 permutations on Bray-Curtis dissimilarity matrices, and explained variance (R^2), F-ratio (F), and *p*-value (*p*) results are reported (lines 7-18). The homogeneity of group dispersions was tested with permutational analysis of multivariate dispersions (PERMDISP) with 999 permutations (lines 22-37). The levels of significance are indicated by asterisks ($P \leq 0.05$: *; $P \leq 0.01$: **; $P \leq 0.001$: ***).
